# A Novel Elite-Guided Hybrid Metaheuristic Algorithm for Efficient Feature Selection

**DOI:** 10.3390/biomimetics10110747

**Published:** 2025-11-06

**Authors:** Zichuan Chen, Bin Fu, Yangjian Yang

**Affiliations:** 1 Faculty of Engineering, The University of Sydney, Sydney, NSW 2006, Australia; zche4427@uni.sydney.edu.au; 2 Taizhou Institute of Zhejiang University, Taizhou 318000, China; fubin@tzizju.cn

**Keywords:** feature selection, Northern Goshawk Optimization, meta-heuristic algorithm, vertical crossover mutation, exploration-exploitation

## Abstract

Feature selection aims to identify a relevant subset of features from the original feature set to enhance the performance of machine learning models, which is crucial for improvig model accuracy. However, this task is highly challenging due to the enormous search space, often requiring the use of meta-heuristic algorithms to efficiently identify near-optimal feature subsets. This paper proposes an improved algorithm based on Northern Goshawk Optimization (NGO), called Elite-guided Hybrid Northern Goshawk Optimization (EH-NGO), for feature selection tasks. The algorithm incorporates an elite-guided strategy within the NGO framework, leveraging information from elite individuals to direct the population’s evolutionary trajectory. To further enhance population diversity and prevent premature convergence, a vertical crossover mutation strategy is adopted, which randomly selects two different dimensions of an individual for arithmetic crossover to generate new solutions, thereby improving the algorithm’s global exploration capability. Additionally, a boundary control strategy based on the global best solution is introduced to reduce ineffective searches and accelerate convergence. Experiments conducted on 30 benchmark functions from the CEC2017 and CEC2022 test set demonstrate the superiority of EH-NGO in global optimization, outperforming eight compared state-of-the-art algorithms. Furthermore, a novel feature selection method based on EH-NGO is proposed and validated on 22 datasets of varying scales. Experimental results show that the proposed method can effectively select feature subsets that contribute to improved classification performance.

## 1. Introduction

In the era of deep integration between big data and artificial intelligence, machine learning models have been widely applied in critical domains such as medical diagnosis, financial risk control, image recognition, and natural language processing [1,2]. The performance of these models is closely tied to the quality of input features. However, raw data often contain a large number of redundant, irrelevant, or even noisy features. Such features not only give rise to the “curse of dimensionality”—leading to longer training times and soaring computational costs—but also interfere with the model’s ability to capture essential data patterns. This may further cause overfitting, where the model performs well on the training set but fails to generalize effectively to unseen test data [3,4]. Against this backdrop, feature selection has emerged as a fundamental data preprocessing technique. Its goal is to extract a representative and discriminative subset of features from the high-dimensional raw feature space, retaining key information while eliminating redundancy and noise. By doing so, it can improve classification accuracy and generalization ability, simplify model structure, and enhance interpretability. Consequently, feature selection has become an indispensable component of the machine learning pipeline and plays a vital role in advancing intelligent systems toward higher efficiency and reliability [5,6,7,8].

Nevertheless, solving the feature selection problem remains highly challenging. In essence, it is a combinatorial optimization problem in which the search space expands exponentially with the number of features. For instance, when a dataset contains 40 features, the number of possible feature subsets already exceeds one trillion (excluding the empty set). Traditional approaches, such as brute-force search and greedy algorithms, are either computationally infeasible due to their excessive complexity or prone to being trapped in local optima, thereby failing to identify the global optimum.

Against this backdrop, metaheuristic algorithms have become mainstream tools for tackling feature selection, owing to their independence from strict mathematical assumptions and their ability to efficiently approximate optimal solutions within vast search spaces [9,10]. Inspired by natural phenomena or biological behaviors, these algorithms typically simulate swarm intelligence to maintain a dynamic balance between “exploration” and “exploitation”. Exploration seeks to broadly investigate unexplored regions of the solution space to avoid missing global optima, while exploitation focuses on refining promising regions to improve solution accuracy [11,12]. Their effectiveness in diverse optimization problems has led to widespread application in feature selection. The advantages of metaheuristic algorithms in this context include reducing feature dimensionality and improving model performance. For example, Song et al. [13] proposed a hybrid feature selection method that integrates correlation-guided clustering with particle swarm optimization (PSO) for high-dimensional data. Hu et al. [14] developed a fuzzy multi-objective PSO-based feature selection approach. Zhou et al. [15] introduced a slime mould algorithm-based method that combines local dimension mutation with global neighborhood search. Tubishat et al. [16] proposed a dynamic butterfly optimization algorithm (DBOA), an enhanced variant of the butterfly optimization algorithm (BOA), for feature selection. Ying Hu et al. [17] investigated a fuzzy multi-objective PSO-based method, PSOMOFS, which establishes fuzzy dominance relations to evaluate candidate particles and employs a fuzzy crowding distance metric to refine the elite archive and determine global leaders. Wen Long et al. [18] developed an improved version of BOA to address high-dimensional feature selection and fault diagnosis in wind turbine systems. Gang Hu et al. [19] proposed an enhanced version of the Black Widow Optimization algorithm, termed SDABWO, for feature selection. Guan Yang et al. [20] introduced an evolutionary Q-learning-based feature selection optimization algorithm (EQL-FS), which leverages reinforcement learning and the global exploration capabilities of PSO within a multi-agent framework. Yu-Peng Chen et al. [21] presented two novel Bacterial Foraging Optimization (BFO) algorithms—the Adaptive Chemotaxis BFO (ACBFO) and the Improved Swarm and Elimination-Dispersal BFO (ISEDBFO)—for feature selection. Finally, Kiana et al. [22] proposed a multi-objective Beluga Whale Optimization (MOBWO) algorithm tailored for feature selection problems.

Although numerous algorithms have been proposed to address feature selection, existing metaheuristic approaches still exhibit notable limitations in this task. For example, some algorithms, such as the standard Particle Swarm Optimization (PSO) [23], suffer from slow convergence due to the lack of guided exploration. Others, such as the basic Grey Wolf Optimizer (GWO) [24], tend to experience rapid loss of population diversity, leading to premature convergence. In addition, certain boundary-handling strategies—such as simply resetting out-of-bound solutions to the boundary—are overly passive. This not only wastes computational resources but also discards valuable search direction information, making it difficult to adapt to high-dimensional and complex feature selection scenarios [25].

Northern Goshawk Optimization (NGO), an intelligent optimization algorithm inspired by the predatory behavior of northern goshawks, has attracted increasing attention due to its simplicity and ease of implementation [26]. NGO possesses a basic balance between exploration and exploitation, and its concise mathematical model makes it easy to implement. It has already demonstrated promising performance in certain low-dimensional optimization problems [27,28]. However, like many other algorithms, NGO still suffers from several issues, including an imbalance between exploration and exploitation, slow convergence, susceptibility to local optima, and limited convergence accuracy.

When applied to complex scenarios such as high-dimensional feature selection, the shortcomings of the standard NGO become more pronounced. In the exploration phase, position updates rely entirely on randomly selected individuals, leading to blind search directions and preventing the algorithm from leveraging high-quality solutions within the population. This often results in slow convergence. In the exploitation phase, perturbations are generated solely through random scaling of an individual’s own position, which lacks an effective mechanism to break similarity among individuals. Consequently, once the population becomes trapped in a local optimum, the algorithm struggles to escape, often causing premature convergence. Moreover, its boundary-handling strategy adopts a “penalization” approach by directly resetting out-of-bound individuals to the boundary. This interrupts the natural evolutionary trajectory of solutions and may cause the algorithm to miss optimal solutions near the boundary, thereby wasting computational resources [29,30,31,32]. These limitations significantly hinder the potential of NGO in feature selection tasks, highlighting the urgent need for targeted improvements to enhance its optimization performance.

To address the aforementioned limitations of the standard Northern Goshawk Optimization (NGO) algorithm and to develop a more efficient optimization method tailored for feature selection tasks, this paper proposes an improved algorithm—Elite-guided Hybrid Northern Goshawk Optimization (EH-NGO). The algorithm retains the core two-stage framework of NGO while introducing three complementary enhancement strategies to comprehensively improve exploration efficiency, exploitation accuracy, and search stability. First, an elite-guided search mechanism is implemented by constructing an “elite pool” composed of the fittest individuals in the population. A dual-selection approach—comprising a 10% probability of taking the average position of elites and a 90% probability of randomly selecting a single elite—is used to determine the guiding direction, providing clear and high-quality search guidance during the exploration phase and accelerating convergence. Second, a vertical crossover mutation strategy is applied, whereby two different dimensions of an individual are randomly selected and linearly combined to generate a new solution. This not only breaks the similarity among population members but also enhances local exploitation while maintaining population diversity, helping the algorithm escape from local optima. Third, the traditional passive penalty-based boundary handling is replaced with a global best-guided boundary control strategy: when an individual exceeds the search boundary, it is actively guided toward a favorable region based on the global best solution. This approach corrects boundary violations while preserving search direction information, thereby reducing ineffective exploration. The improvements of EH-NGO compared with the original NGO are shown in Table 1.

To validate the effectiveness of the EH-NGO algorithm, systematic experiments were conducted from two perspectives: global optimization performance and feature selection applications. On one hand, the global optimization capability of EH-NGO was evaluated on 30 benchmark functions from the CEC2017 and CEC2022 test suite, which cover a variety of characteristics, including unimodal, multimodal, and high-dimensional functions. EH-NGO was compared with eight state-of-the-art metaheuristic algorithms—VPPSO, IAGWO, LSHADE-cnEpSin, LSHADE-SPACMA, CPO, BKA, NRBO, and the original NGO—considering convergence speed, solution accuracy, and stability. On the other hand, a feature selection method based on EH-NGO, combined with the k-nearest neighbors (KNN) classifier (denoted as EH-NGO-KNN), was tested on 18 datasets of varying sizes. Using “classification error rate + feature subset size” as the optimization objective, the experiments assessed the algorithm’s ability to balance the reduction in feature numbers with the improvement of classification performance.

The main contributions of this paper are as follows:

(1) An Elite-guided Hybrid Northern Goshawk Optimization algorithm (EH-NGO) is proposed, integrating three strategies: elite-guided search, vertical crossover mutation, and global-best guided boundary control.
(2) Experiments on the CEC2017 and CEC2022 test suite show that EH-NGO performs better in population diversity and exploration–exploitation balance. Ablation studies confirm the effectiveness of the three strategies, and comparative experiments with eight mainstream algorithms validate the superiority of EH-NGO.
(3) EH-NGO is applied to feature selection, and the EH-NGO-KNN framework is proposed. By integrating the KNN classifier and optimizing “classification error rate + subset size”, the method achieves a balance between “reducing features” and “improving model performance,” providing an efficient solution for high-dimensional feature selection.

The remainder of this paper is organized as follows: Section 2 details the basic principles of the original NGO algorithm, the design of the three enhancement strategies of EH-NGO, as well as the pseudocode and time complexity analysis. Section 3 presents numerical experiments based on the CEC2017 and CEC2022 test suite. Section 4 focuses on the application of EH-NGO to feature selection, including the mathematical modeling of the problem, the EH-NGO-KNN framework, and experiments on multiple datasets. Section 5 summarizes the findings and outlines future research directions.

## 2. Northern Goshawk Optimization and the Proposed Methodology

### 2.1. Northern Goshawk Optimization

The fundamental principle of the Northern Goshawk Optimization (NGO) algorithm is to simulate the hunting strategy of northern goshawks, which consists of two stages: prey detection and attack (exploration phase) and pursuit and evasion (exploitation phase). In the first stage, the goshawk randomly selects a prey and swiftly attacks it, enhancing the exploration capability of the NGO algorithm by performing a global search across the solution space to identify promising regions. In the second stage, after the initial attack, the prey attempts to escape, requiring the goshawk to continue the pursuit. This behavior simulation improves the algorithm’s local search ability within the solution space [26].

#### 2.1.1. Initialization

The NGO method is a population-based metaheuristic algorithm, similar to other heuristic approaches. Like other such algorithms, NGO randomly generates a set of candidate solutions within the search space:

(1) $$X={\left[\begin{array}{c} X_{1}\\ X_{2}\\ \vdots \\ X_{i}\\ \vdots \\ X_{pop} \end{array}\right]}_{pop\times dim}={\left[\begin{array}{cccccc} x_{1,1} & x_{1,2} & \cdots & x_{1,j} & \cdots & x_{1,dim}\\ x_{2,1} & x_{2,2} & \cdots & x_{2,j} & \cdots & x_{2,dim}\\ \vdots & \vdots & \ddots & \vdots & \ddots & \vdots \\ x_{i,1} & x_{i,2} & \cdots & x_{i,j} & \cdots & x_{i,dim}\\ \vdots & \vdots & \ddots & \vdots & \ddots & \vdots \\ x_{pop,1} & x_{pop,2} & \cdots & x_{pop,j} & \cdots & x_{pop,d} \end{array}\right]}_{pop\times dim}$$

where X denotes the population of northern goshawks, $X_{i}$ represents the position of the $i^{th}$ goshawk, and $x_{i,j}$ represents the position of the $j^{th}$ problem variable for the $i^{th}$ goshawk. The parameters pop and dim correspond to the population size and the dimensionality of the problem, respectively. The initial positions of the goshawks are randomly determined according to the following equation:

(2) $$X=\left(ub-lb\right)\cdot rand+lb$$

where X represents the initial value of the candidate solution variables, ub and lb denote the upper and lower bounds, respectively, and rand is a uniformly distributed random number in the range (0, 1).

#### 2.1.2. Exploration (Attack Prey)

In the first stage of the northern goshawk’s hunting behavior, a prey is randomly selected and swiftly attacked. Since the choice of prey in the search space is random, this stage enhances the exploration capability of the NGO algorithm. During this phase, the goshawk performs a global search across the solution space with the goal of identifying promising regions. The mathematical model for this stage is described by Equations (3) and (4) [26]:

(3) $$\begin{array}{c} X\left(t+1\right)=\left\{\begin{array}{l} X\left(t\right)+rand\cdot \left(P\left(t\right)-I\cdot X\left(t\right)\right),\;f\left(P\left(t\right)\right)<f\left(X\left(t\right)\right)\\ X\left(t\right)+rand\cdot \left(X\left(t\right)-P\left(t\right)\right),\;f\left(P\left(t\right)\right)\geq f\left(X\left(t\right)\right) \end{array}\right. \end{array}$$

(4) $$\begin{array}{c} X_{new}\left(t+1\right)=\left\{\begin{array}{cc} X\left(t+1\right), & if\;f\left(X\left(t+1\right)\right)<f\left(X\left(t\right)\right)\\ X\left(t\right), & if\;f\left(X\left(t+1\right)\right)\geq f\left(X\left(t\right)\right) \end{array}\right. \end{array}$$

where $X\left(t+1\right)$ and $X\left(t\right)$ represent the position information of the Northern Goshawk at the next iteration and the current iteration, respectively. $X_{new}$ denotes the updated state of the goshawk in the first stage, rand represents a 1×dim array of random numbers uniformly generated from the interval [0, 1], I is a random integer taking the value 1 or 2, and $f\left(\cdot \right)$ represents the fitness value of the objective function. RB denotes a 1×dim array generated from a standard normal distribution (mean 0, standard deviation 1), and P represents the position of the prey.

#### 2.1.3. Exploitation (Chase Prey)

During the prey pursuit stage, after the northern goshawk attacks its prey, the prey attempts to escape while the goshawk continues the chase. Due to the goshawk’s high pursuit speed, it can nearly always catch the prey under any circumstance. Simulating this behavior enhances the algorithm’s local search capability within the solution space. The mathematical model for the second stage is expressed by Equations (5)–(7) [26]:

(5) $$\begin{array}{c} X\left(t+1\right)=X\left(t\right)+R\cdot \left(2\cdot rand-1\right)\cdot X\left(t\right) \end{array}$$

(6) $$\begin{array}{c} R=0.02\times \left(1-\frac{t}{T}\right) \end{array}$$

(7) $$\begin{array}{c} X_{new}\left(t+1\right)=\left\{\begin{array}{cc} X\left(t+1\right), & if\;f\left(X\left(t+1\right)\right)<f\left(X\left(t\right)\right)\\ X\left(t\right), & if\;f\left(X\left(t+1\right)\right)\geq f\left(X\left(t\right)\right) \end{array}\right. \end{array}$$

where rand denotes a 1×dim array of random numbers generated from a normal distribution, t is the current iteration, T is the maximum number of iterations, and $X_{new}\left(t+1\right)$ represents the updated position of the goshawk.

### 2.2. Proposed EH-NGO

#### 2.2.1. Elite-Guided Search Strategy

**Figure 1:**
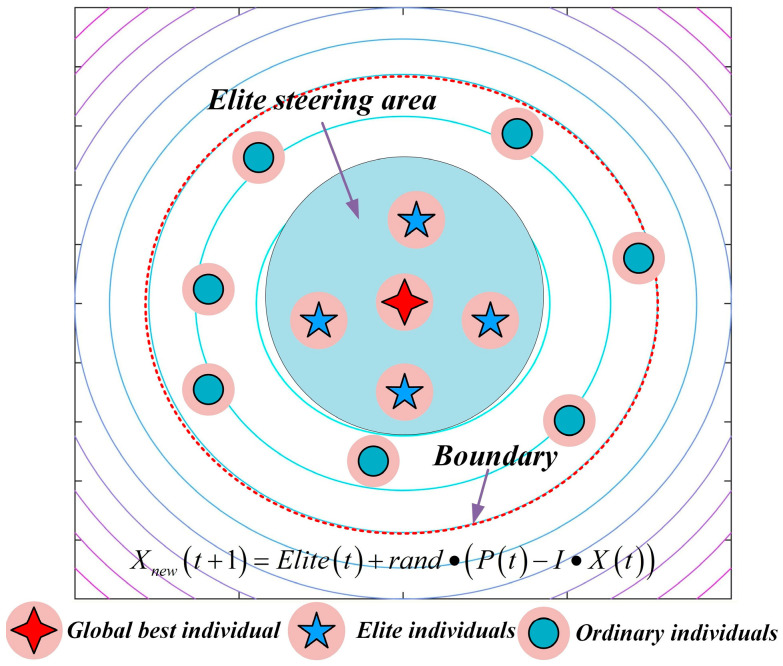
Schematic diagram of elite-guided search strategy.

In the standard NGO algorithm, position updates during the exploration phase rely entirely on randomly selected individuals, which introduces a high degree of blind search. Although this mechanism helps maintain population diversity, it does not exploit high-quality search directions, resulting in slow convergence and difficulty in consistently exploring the most promising regions. To enhance the directional guidance during exploration, the concept of an “elite pool” is introduced. In each iteration, the top P individuals with the highest fitness values in the population $\left(P=5\right)$ are selected to form the elite pool. A dual-selection mechanism—“10% probability of taking the average position of the elites + 90% probability of randomly selecting a single elite”—is employed to determine the guiding individual, denoted as Elite. The position update formula for the exploration phase is reconstructed as Equation (8) and illustrated in Figure 1, allowing individuals performing worse than randomly selected members to conduct their search based on the elites:

(8) $$\begin{array}{c} X\left(t+1\right)=\left\{\begin{array}{l} Elite\left(t\right)+rand\cdot \left(P\left(t\right)-I\cdot X\left(t\right)\right),\;f\left(P\left(t\right)\right)<f\left(X\left(t\right)\right)\\ X\left(t\right)+rand\cdot \left(X\left(t\right)-P\left(t\right)\right),\;f\left(P\left(t\right)\right)\geq f\left(X\left(t\right)\right) \end{array}\right. \end{array}$$

#### 2.2.2. Vertical Crossover Mutation Strategy

**Figure 2:**
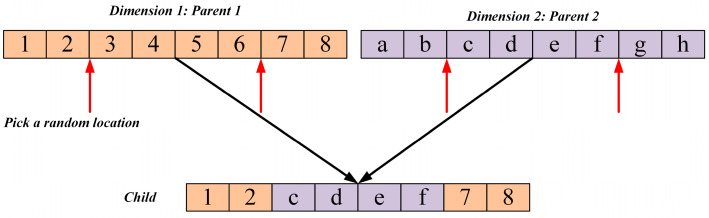
Schematic diagram of the vertical crossover mutation strategy.

In the exploitation phase of the standard NGO algorithm, the perturbation mechanism is relatively simple, relying primarily on random scaling of the individual’s own position, and lacks an effective strategy to escape local optima. Once the population is trapped at a local extremum, this perturbation alone is insufficient to enable individuals to break free, leading to premature convergence. To enhance the algorithm’s local exploitation capability and its ability to escape local optima, a vertical crossover mutation strategy is proposed. As illustrated in Figure 2, to break the similarity among individuals in the population, a “dimension-wise crossover” mutation mechanism is designed: for each individual, two distinct dimensions, $j_{1}$ and $j_{2}$ $\left(j_{1}\neq j_{2}\right)$, are randomly selected, and a new dimension value is generated through a linear combination as follows (Equation (9)), while the remaining dimensions remain unchanged. The resulting new individual is then evaluated and selected based on fitness:

(9) $$\begin{array}{c} X_{vc}\left(j_{1}\right)=\alpha \cdot X\left(i,j_{1}\right)+\left(1-\alpha \right)\cdot X\left(i,j_{2}\right) \end{array}$$

where α is a random number in the range [0, 1].

This strategy recombines information from different dimensions within a single individual, generating novel solutions in its neighborhood. The dimension-wise crossover effectively exploits the evolutionary potential of the individual, introduces diversity during fine-grained search, helps the algorithm escape from local optima, and improves solution accuracy.

#### 2.2.3. Global-Best Guided Boundary Control Strategy

The standard NGO algorithm typically employs simple boundary-handling methods, such as directly resetting individuals to the boundary. This approach interrupts the natural trajectory of individuals, causing them to lose search direction information from the current generation and represents a passive “penalty” measure. Such handling not only wastes computational resources but may also prevent the population from exploring potentially optimal regions near the boundaries.

**Figure 3:**
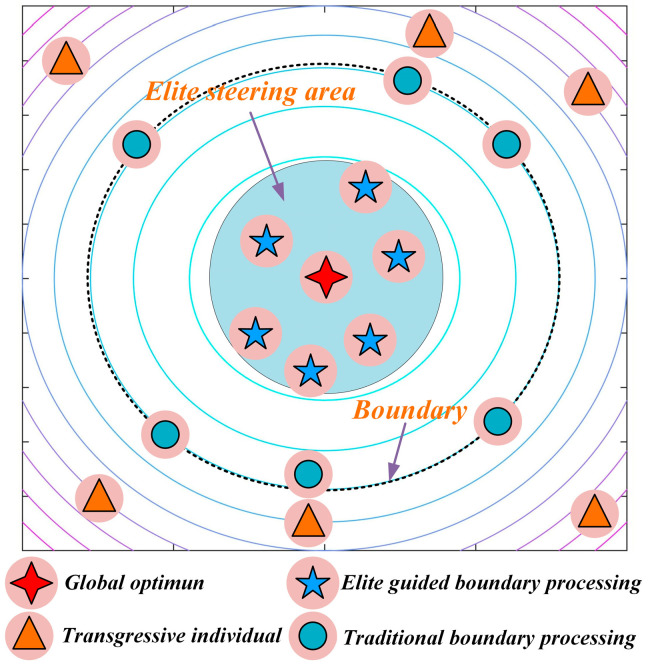
Schematic diagram of global-best guided boundary control strategy.

To transform boundary handling from a passive “penalty” into an active “guidance” mechanism, a global best-guided boundary control strategy is proposed. As shown in Figure 3, when an individual exceeds the boundary, instead of simply resetting it, the strategy guides the individual, with a certain probability, toward the region near the current global best solution. This approach turns a failed boundary exploration into an effective exploitation of high-quality regions, correcting the boundary violation while fully utilizing historical search information, thereby significantly improving search efficiency. The strategy is mathematically expressed as follows (Equation (10)):

(10) $$\begin{array}{c} X\left(i,j\right)=\left\{\begin{array}{c} X_{best}\left(j\right)+0.5\times rand\times \left(ub\left(j\right)-X_{best}\left(j\right)\right),\;if\;X\left(i,j\right)\;>\;ub\left(j\right)\\ X_{best}\left(j\right)-0.5\times rand\times \left(X_{best}\left(j\right)-lb\left(j\right)\right),\;if\;X\left(i,j\right)\;<\;lb\left(j\right) \end{array}\right. \end{array}$$

Based on the above discussion, the pseudocode for EH-NGO is presented in Algorithm 1.
**Algorithm 1.** Pseudo-Code of EH-NGO.*1: Initialize Problem Setting (population*$\left(pop\right),\;dim$, ub, lb*), Max iterations*$\left(T\right)$. *2: Initialize a set of Northern Goshawk’ (*$X_{i}(i=1,2,\ldots pop)$*).* *3: **while*** t=1:T ***do*** *4:   Calculate the fitness of the population.* *5:   **Exploration:*** ***Attack prey*** *6:    Calculate the fitness* $X\left(t+1\right)$ *using Equation (8).* *7:    Update the position of the current individual using Equation (4).* *8:    Using Equation (10) for boundary adjustment.* *9:  **Exploitation:*** ***Chase prey*** *10:    Calculate the fitness* $X\left(t+1\right)$ *using Equations (5) and (6).* *11:    Update the position of the current individual using Equation (7).* *12:    Using Equation (10) for boundary adjustment.* *13:  **End for*** *14:  Calculate new position of the current individual using Equation (9).* *15:  Update the best solution found so far* $X_{best}$. *16: **End while*** *17: Return* $X_{best}$.

### 2.3. Computational Time Complexity of EH-NGO

The performance of an algorithm is crucial, but it is equally important to evaluate its time complexity. In many optimization tasks, an algorithm must not only demonstrate excellent performance but also exhibit high real-time efficiency. Time complexity reflects how the algorithm’s runtime scales with the size of the input. Analyzing the time complexity of an optimization algorithm helps estimate its computational cost when handling large-scale problems. In the standard NGO, the computational complexity of the defined control parameters is O(N×dim), where N represents the population size and dim denotes the problem dimension. During the initialization phase, the algorithm requires O(N×dim) time. Furthermore, over T iterations, the computational complexity for updating individual positions is O(T×N×dim). Therefore, the overall computational complexity of the NGO algorithm can be expressed as O(T×N×dim). n the proposed EH-NGO, since only the position update strategy and the objective function evaluation method are improved without introducing additional complexity factors, the time complexity remains O(T×N×dim).

## 3. Numerical Experiments

### 3.1. Algorithm Parameter Settings

In this section, the performance of the proposed EH-NGO algorithm is evaluated using the most challenging numerical optimization benchmark suite, CEC2017, and compared with several other algorithms. The comparison algorithms include: Velocity Pausing Particle Swarm Optimization (VPPSO) [33], Improved multi-strategy adaptive Grey Wolf Optimization (IAGWO) [34], LSHADE-cnEpSin [35], LSHADE-SPACMA [36], Crested Porcupine Optimizer (CPO) [37], Black-winged Kite Algorithm (BKA) [38], Neighbor Regularized Bayesian Optimization (NRBO) [39], and Northern Goshawk Optimization (NGO) [26]. The algorithm’s parameters are listed in Table 2.

### 3.2. Qualitative Analysis of EH-NGO

#### 3.2.1. Analysis of the Population Diversity

In optimization algorithms, population diversity refers to the extent of differences among individuals within a population [40,41], with each individual generally representing a candidate solution. A reduction in diversity often results in premature convergence to local optima, which can limit the algorithm’s ability to explore the global search space. Conversely, maintaining greater diversity supports broader exploration of potential solutions and increases the likelihood of identifying the global optimum. In this section, we assess the population diversity of the EH-NGO approach using Equation (11) [11,42].

(11) $$I_{C}\left(t\right)=\sqrt{\sum_{i=1}^{N}\sum_{d=1}^{D}{\left(x_{id}\left(t\right)-c_{d}\left(t\right)\right)}^{2}},$$

where $I_{C}\left(t\right)$ denotes the population diversity, N represents the population size, D indicates the problem’s dimensionality, and $x_{id}\left(t\right)$ denotes the value of the i individual in the d dimension at the t iteration. $c_{d}\left(t\right)$ quantifies the dispersion degree of the entire population relative to its center of mass at iteration t, which is calculated using Equation (13).

(12) $$c_{d}\left(t\right)=\frac{1}{D}\sum_{i=1}^{N}x_{id}\left(t\right).$$

**Figure 4:**
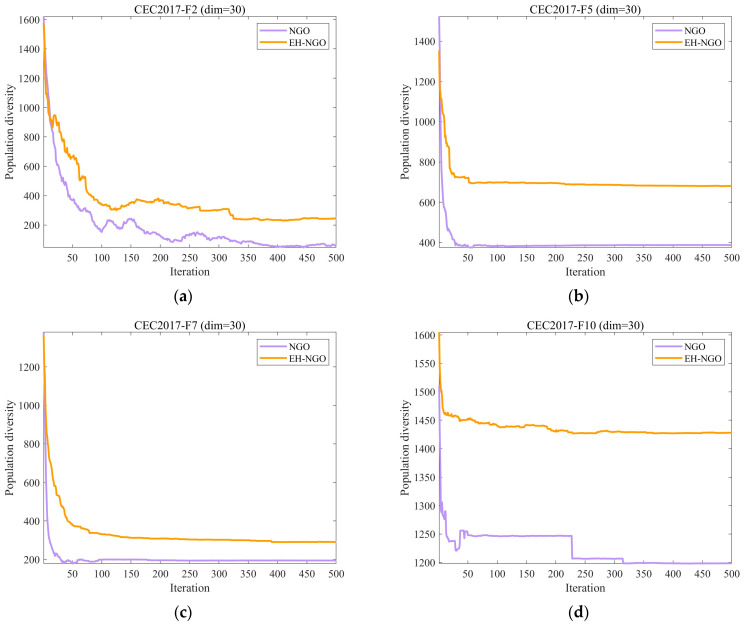

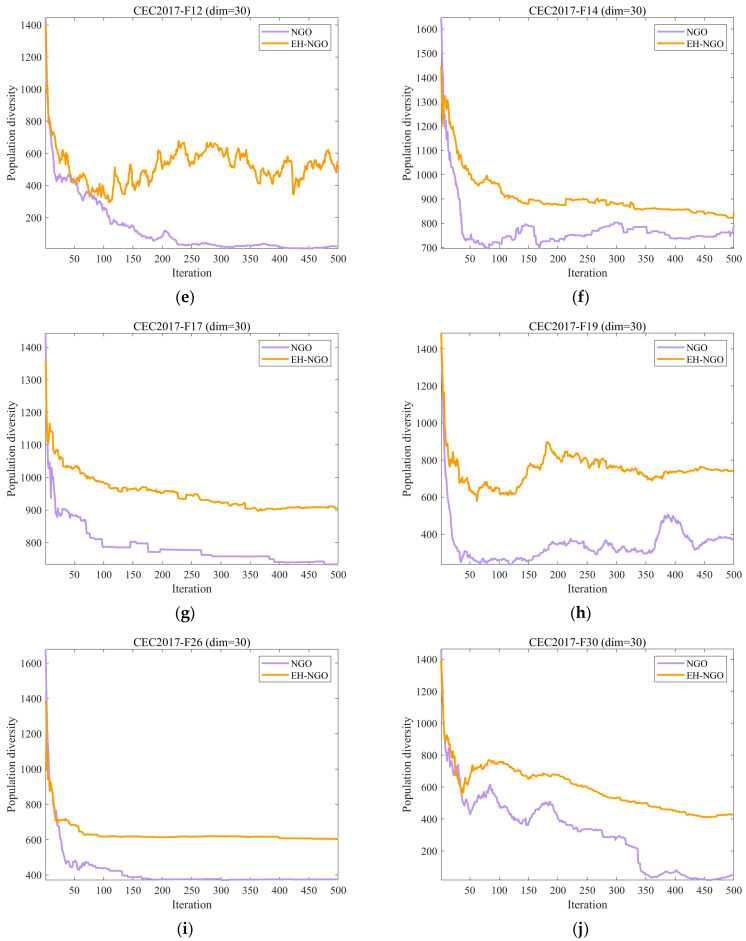
The analysis of the population diversity of NH-NGO and NGO.

Figure 4 presents the population diversity evolution curves of EH-NGO and the original NGO algorithm on the CEC2017 benchmark suite. The population diversity metric $I_{C}\left(t\right)$ reflects the dispersion of individuals in the solution space during the iterations, with its magnitude directly related to the algorithm’s global exploration capability and the risk of premature convergence—excessively high diversity may reduce search efficiency, while overly low diversity can lead to stagnation in local optima. As shown in the figure, during the initial iterations (the first 50 iterations), both algorithms maintain relatively high diversity levels, indicating strong global search ability capable of broadly exploring the solution space. However, differences gradually emerge as the iterations progress (50–200 iterations). The diversity curve of the original NGO declines more rapidly, and for most functions (e.g., F2, F7, F12), it enters a low-diversity stable state earlier. For example, after 200 iterations on the F2 function, the diversity of NGO drops below 600, whereas EH-NGO maintains a value above 800, reflecting a rapid increase in population similarity and a higher risk of premature convergence. In contrast, EH-NGO, benefiting from the vertical crossover mutation strategy that recombines dimensional information within individuals and the elite-guided strategy that optimizes search directions, consistently maintains higher diversity levels than NGO, with a more gradual decline. Even in the later iterations (300–500), EH-NGO preserves a certain level of diversity; for instance, after 500 iterations on the F10 function, EH-NGO’s diversity is approximately 700, compared to around 400 for NGO. These results demonstrate that the introduction of the proposed strategies in EH-NGO effectively enhances the maintenance of population diversity while ensuring search efficiency, providing crucial support for escaping local optima and continuously exploring global optima. This advantage is particularly pronounced in high-dimensional complex functions such as F12 and F30.

#### 3.2.2. Analysis of the Exploration and Exploitation

In optimization algorithms, both exploration and exploitation play crucial roles. Exploration involves the broad search across different regions of the solution space to discover new areas that may contain the global optimum. Exploitation, on the other hand, focuses on refining and improving existing high-quality solutions through an intensive local search, leveraging current information to achieve higher precision.

Overemphasis on exploration can cause inefficient allocation of computational resources, as the algorithm may scan extensively without sufficiently improving promising solutions, missing opportunities for local refinement. Conversely, excessive exploitation increases the risk of premature convergence to local optima, limiting the search for better solutions in other regions [9,43]. Hence, achieving an appropriate balance between these two processes is essential for algorithmic performance. In this part, we examine the exploratory and exploitative behaviors of the EH-NGO algorithm, as measured by Equations (13) and (14) [11].

(13) $$Exploration\left(\%\right)=\frac{Div\left(t\right)}{Div_{max}}\times 100\%,$$

(14) $$Exploitation\left(\%\right)=\frac{\left|Div\left(t\right)-Div_{max}\right|}{Div_{max}}\times 100\%,$$

where $Div\left(t\right)$ denotes the measure of diversity at the tth iteration, which is calculated by Equation (15) and $Div_{max}$ denotes the maximum measure of diversity throughout the iteration.

(15) $$Div\left(t\right)=\frac{1}{D}\sum_{d=1}^{D}\frac{1}{N}\sum_{i=1}^{N}|median\left(x_{d}\left(t\right)\right)-x_{id}\left(t\right)|.$$

**Figure 5:**
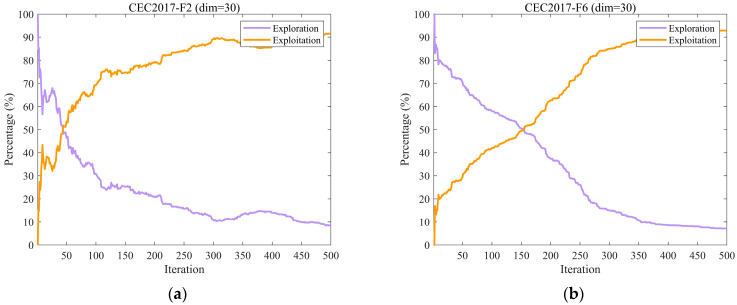

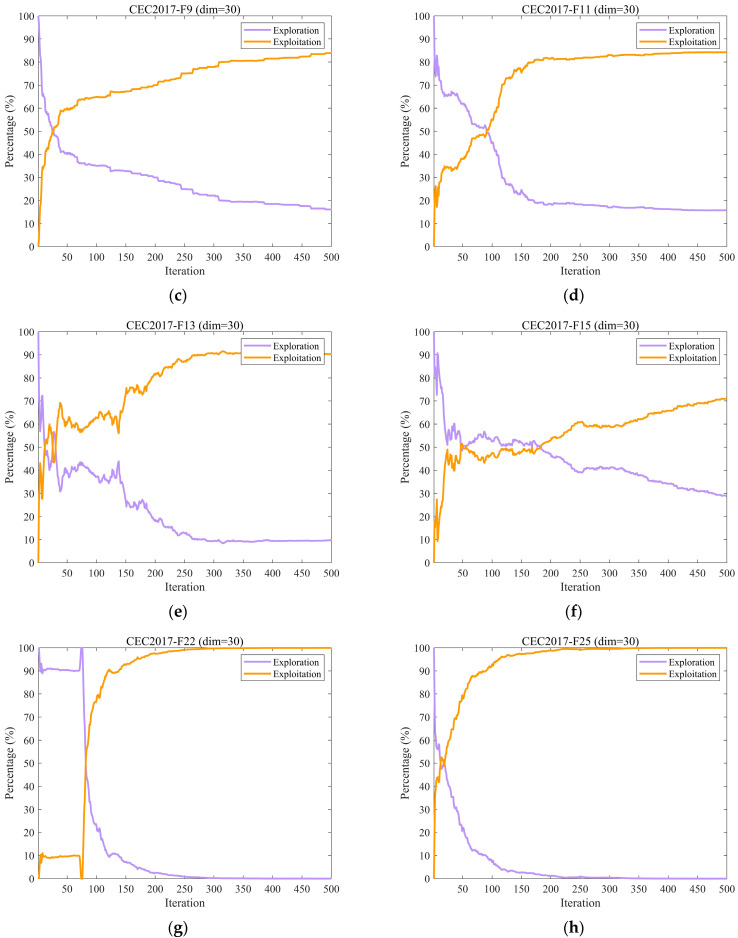

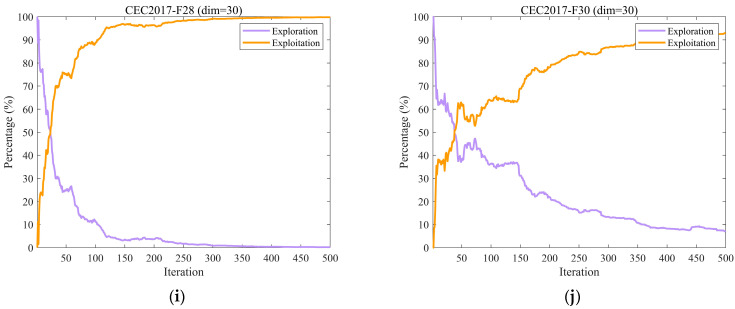
The analysis of the exploration and exploitation of EH-NGO.

Figure 5 illustrates the dynamic changes in exploration and exploitation rates of EH-NGO on ten representative benchmark functions from the CEC2017 test suite (F2, F6, F9, etc.), all with a dimensionality of 30. The exploration rate reflects the algorithm’s capability to globally search unknown regions, while the exploitation rate indicates its ability to perform local optimization around high-quality solutions. The balance between these two factors directly affects algorithm performance. During the initial iterations (the first 50 iterations), EH-NGO maintains an exploration rate of 70–90%, enabling rapid localization of potential optimal regions, which is facilitated by the elite-guided strategy that reduces blind exploration. In the middle stage (50–200 iterations), the exploration rate gradually stabilizes around 40%, while the exploitation rate simultaneously increases, achieving a smooth transition from exploration to exploitation. During this phase, the vertical crossover mutation strategy ensures deep local search while preventing the population from being trapped in local optima. In the later stage (300–500 iterations), the exploration rate stabilizes at 20–30%, allowing exploitation to dominate for solution refinement, while low-intensity exploration is maintained to prevent missing better solutions. Even on complex functions such as F22 and F30, EH-NGO maintains a stable exploration–exploitation balance, demonstrating that the proposed improvement strategies enable dynamic adaptation between global and local search, effectively combining search breadth with exploitation precision.

#### 3.2.3. Impact Analysis of the Strategy

**Figure 6:**
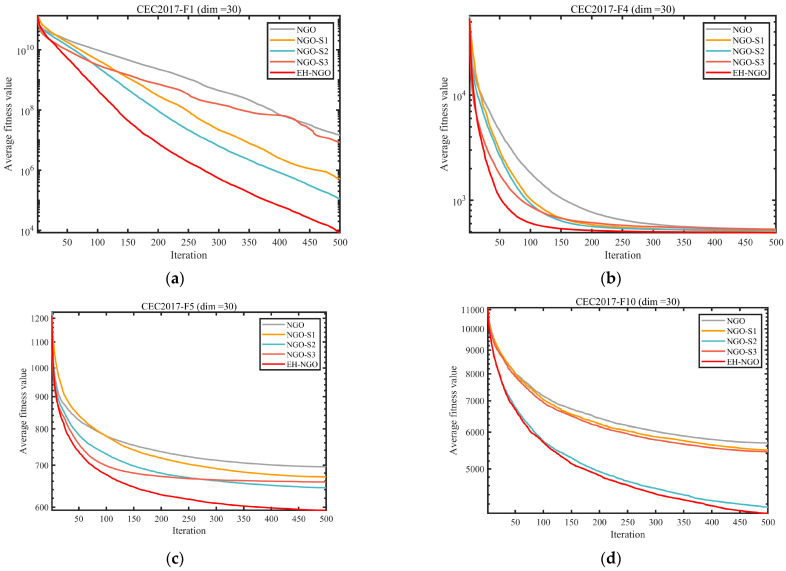

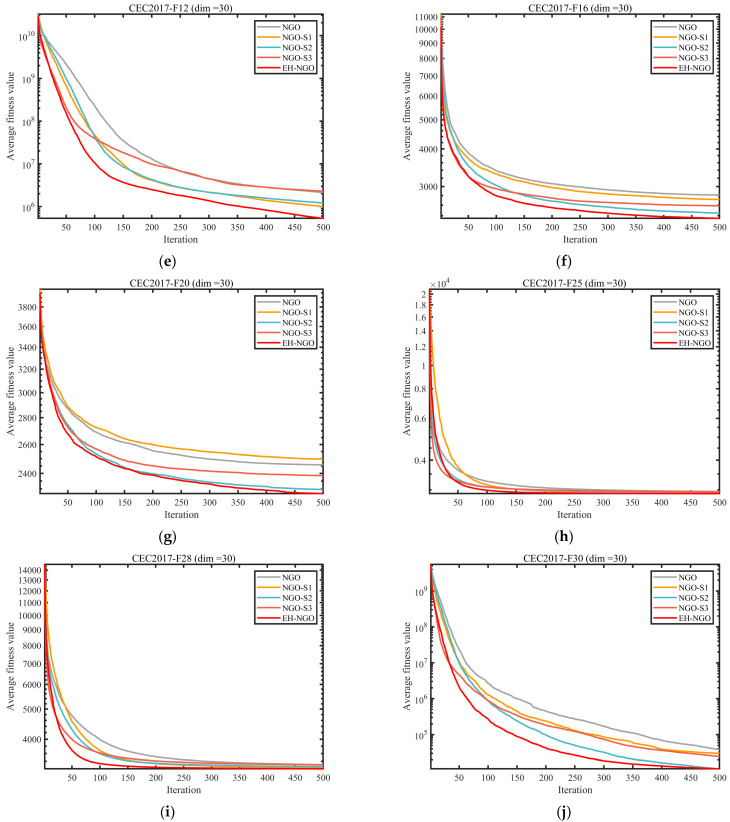
Comparison of different improvement strategies.

**Figure 7:**
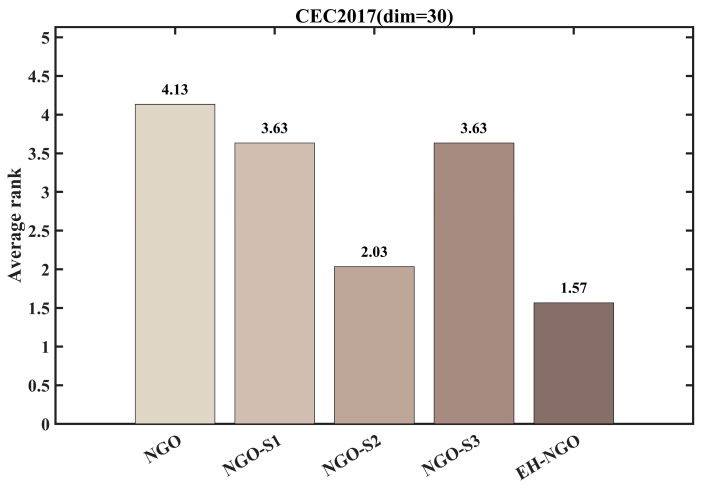
Average ranking of COA improved by different strategies.

To evaluate the individual contributions and synergistic effects of the three enhancement strategies—Elite-guided search strategy (S1), Vertical crossover mutation strategy (S2), and Global-best guided boundary control strategy (S3)—on the benchmark NGO algorithm, ablation experiments were conducted using the CEC2017 test suite with dimensionality dim=30. Five comparative variants were designed: the standard NGO, NGO_S1 (containing only S1), NGO_S2 (containing only S2), NGO_S3 (containing only S3), and EH-NGO integrating all three strategies. The experimental results are shown in Figure 6 and Figure 7.

The convergence curves in Figure 6 indicate that each individual strategy can improve algorithm performance, albeit with varying effects. NGO_S1 (S1 only), guided by elite individuals, exhibits faster convergence in the early stage compared to the standard NGO (e.g., on the F1 function after 100 iterations, the objective value is 1–2 orders of magnitude lower than NGO), but is prone to being trapped in local optima later. NGO_S2 (S2 only), through dimension-wise crossover mutation, enhances local search ability and is able to escape the local extrema of NGO on multimodal functions (e.g., F10, F28), significantly improving convergence precision. NGO_S3 (S3 only), guided by global-best boundary control, reduces ineffective searches and performs better than the single S1 or S2 variants on boundary-optimal problems (e.g., F5, F16). When all three strategies are integrated, EH-NGO achieves convergence curves consistently below all comparative variants. For example, on functions such as F1 and F12 after 500 iterations, the objective values of EH-NGO are 1–3 orders of magnitude lower than the best single-strategy variant (NGO_S2), with improved convergence stability (smaller fluctuations in the curves).

The average ranking results in Figure 7 further quantify the value of each strategy. The standard NGO has an average rank of approximately 3.63, while among the single-strategy variants, NGO_S2 achieves the best ranking (2.03), followed by NGO_S1 (3.63) and NGO_S3 (1.57). EH-NGO attains an average rank of only 1.57, significantly outperforming all other variants. These results demonstrate a clear synergistic effect among the three strategies: S1 accelerates early convergence, S2 enhances local exploitation and the ability to escape local optima, and S3 improves search efficiency. Their combination enables EH-NGO to achieve both speed and precision in global optimization, validating the rationality and effectiveness of the proposed improvement strategies.

### 3.3. Experimental Results and Analysis of CEC2017 and CEC2022 Test Suite

**Figure 8:**
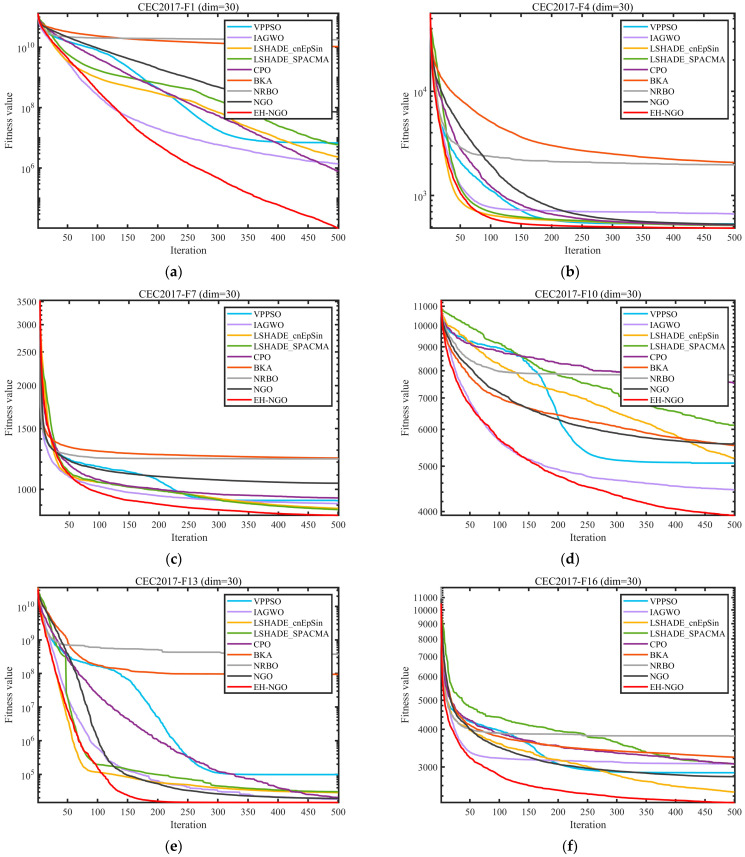

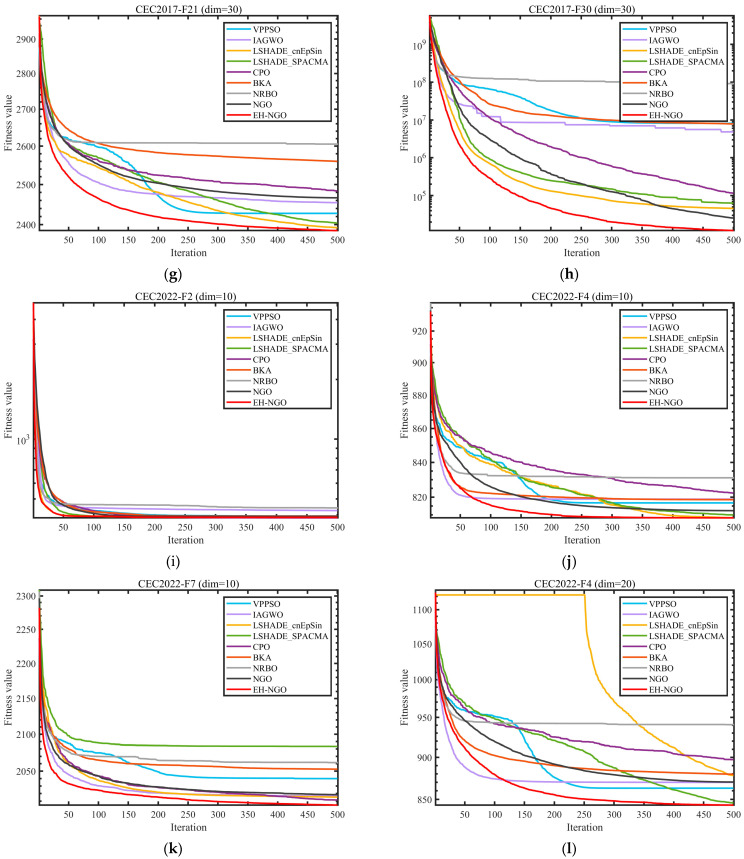

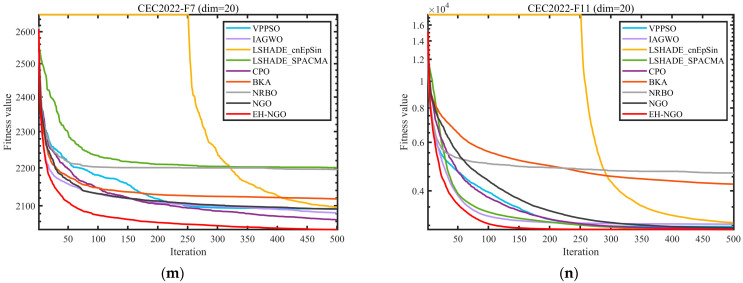
Comparison of convergence speed of different algorithms on CEC2017 test set.

**Figure 9:**
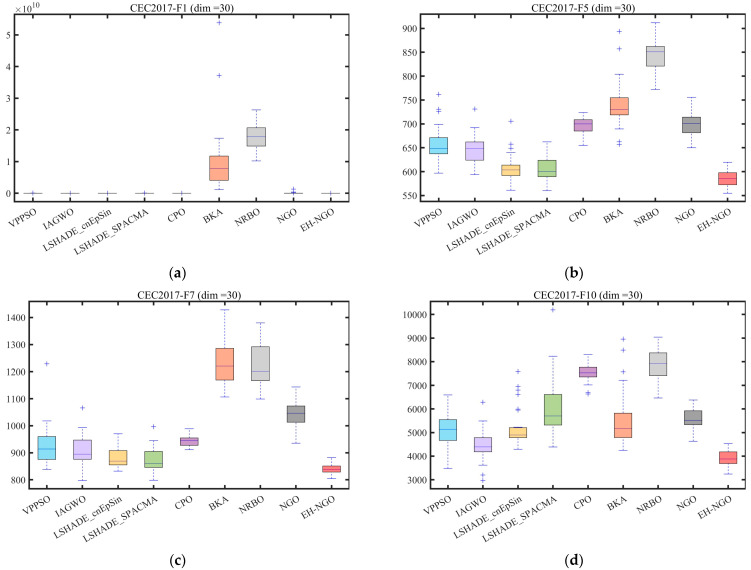

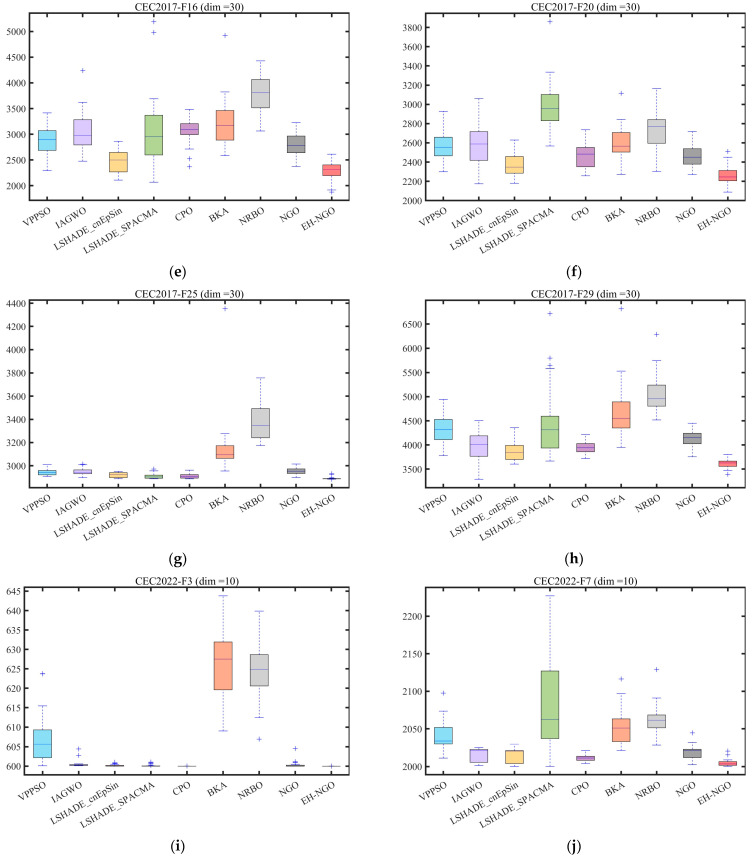

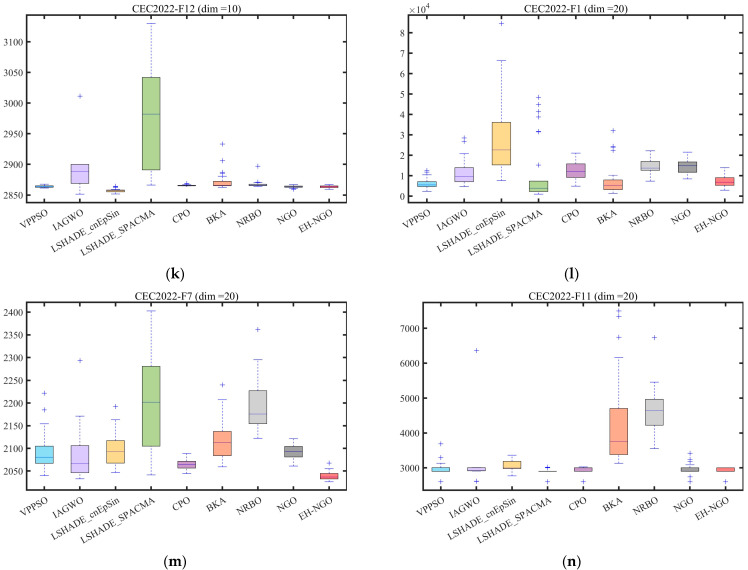
Boxplot analysis for different algorithms on the CEC2017 test set.

This section evaluates the performance of EH-NGO using the CEC2017 and CEC2022 benchmark suite. To comprehensively test its capabilities, experiments were conducted on the CEC2017(dim = 30) and CEC2022(dim = 10/20) functions, and the experimental settings are summarized in Table 3, Table 4 and Table 5. The parameter configurations for all algorithms are listed in Table 1. To ensure fairness and mitigate randomness, a constant population size of 30 and a maximum of 500 iterations were employed for all algorithms. Each algorithm was independently executed 30 times, and the mean (Ave), standard deviation (Std), and ranking (Rank) were recorded, with the best results highlighted in bold. All experiments were performed in a Windows 11 environment with an AMD Ryzen 7 9700X 8-Core Processor (3.80 GHz), 48 GB RAM, using MATLAB 2024b. The convergence curves and boxplots of the different algorithms are presented in Figure 8 and Figure 9, respectively.

To validate the global optimization performance of EH-NGO, systematic experiments were conducted on the CEC2017 (30-dimensional) and CEC2022 (10-dimensional, 20-dimensional) test sets, and comparisons were made with eight mainstream metaheuristic algorithms, including VPPSO, IAGWO, and LSHADE-cnEpSin. The experimental results are shown in Table 2, Table 3 and Table 4. According to the statistical indicators, EH-NGO demonstrates superior convergence accuracy and stability on most functions: in the 30-dimensional functions of the CEC2017 test set, the average objective value (Ave) of EH-NGO on 18 functions, such as F1, F4, and F12, is significantly lower than that of the comparison algorithms. Specifically, the Ave value of the F1 function is reduced by 1–2 orders of magnitude compared to the original NGO, while the Ave value of the F12 function is approximately 30% lower than that of the second-best performing LSHADE-SPACMA. Additionally, EH-NGO achieves the smallest standard deviation (Std) on most functions. For example, the Std value of the F7 function is only 8.4076, which is far lower than VPPSO’s 9.2783 and IAGWO’s 9.0957, indicating stronger stability in its optimization results.

In different dimensional scenarios of the CEC2022 test set, the advantages of EH-NGO become even more pronounced: in the 10-dimensional scenario, it achieves theoretical optimal solutions on functions such as F3, F5, and F9 (e.g., for the F3 function, Ave = 6.0000, Std = 2.5408 × 10^−3^). Moreover, on complex multimodal functions like F7 and F11, the Ave values are reduced by 5–15% compared to the comparison algorithms. In the 20-dimensional high-dimensional scenario, EH-NGO maintains its leading performance on functions such as F2, F4, and F10. For instance, the Ave value of the F2 function is 4.4771 × 10^3^, which is approximately 5.4% lower than the second-best algorithm LSHADE-cnEpSin (4.7355 × 10^2^), and the Std value is only 1.8899, demonstrating its ability to mitigate optimization accuracy degradation in high-dimensional spaces.

Figure 8 shows the convergence curves of different algorithms on representative functions of CEC2017 and CEC2022, allowing for a visual observation of EH-NGO’s convergence efficiency advantage. On the F1 function (30-dimensional) of CEC2017, EH-NGO enters the objective value range of 10^5^ after just 50 iterations, while the original NGO requires over 150 iterations to reach a similar level. Algorithms such as VPPSO and IAGWO struggle to break through the 10^6^ range. On the F30 function (high-dimensional complex function) of CEC2017, EH-NGO’s convergence curve consistently lies below all comparison algorithms. After 500 iterations, its objective value is approximately 60% lower than that of the original NGO and about 75% lower than algorithms like BKA and NRBO, indicating that its global exploration capability effectively handles high-dimensional complex search spaces. On the F4 function (10-dimensional, 20-dimensional) of CEC2022, EH-NGO exhibits a rapid decline trend in the early iterations (first 50 iterations) and maintains stable optimization in the later iterations (300–500 iterations), avoiding local stagnation. In contrast, comparison algorithms like LSHADE-SPACMA enter a plateau phase after 200 iterations, with no further improvement in optimization accuracy.

The boxplots in Figure 9 further reveal the distribution characteristics of the algorithm optimization results. Taking the F1 function of CEC2017 as an example, EH-NGO’s boxplot has the smallest box height (approximately 10^4^) and no outliers, indicating extremely low dispersion in its multiple independent runs. In contrast, algorithms like VPPSO and BKA have box heights exceeding 10^5^ and contain numerous outliers, reflecting the instability of their optimization results. On the F10 function (multimodal function) of CEC2017, EH-NGO’s median objective value is approximately 40% lower than that of the original NGO, and the interquartile range is only one-third of that of the original NGO, demonstrating its ability to stably find high-quality solutions even in multi-extremum scenarios. On the F7 function (10-dimensional, 20-dimensional) of CEC2022, EH-NGO’s boxplot consistently occupies the lowest range and exhibits a compact box, further validating its robustness across different dimensional scenarios.

In summary, combining the statistical results from Table 3, Table 4 and Table 5 and the visual analysis from Figure 8 and Figure 9, it is evident that EH-NGO, through the synergistic effects of elite-guided search, vertical crossover mutation, and global optimal boundary control strategies, significantly outperforms the comparison algorithms in convergence accuracy, convergence speed, and stability. It effectively handles complex optimization scenarios such as low-dimensional unimodal and high-dimensional multimodal problems, laying a solid foundation for subsequent feature selection applications.

### 3.4. Friedman Mean Rank Test

In this subsection, the Friedman test [44] is used to determine the overall ranking of the EN-NGO algorithm relative to other methods. As a nonparametric approach, the Friedman test is suitable for comparing median performance differences across three or more matched groups. It is particularly well-suited for repeated measures or block designs, and is often employed as a robust alternative to ANOVA when the assumption of normality is violated. The Friedman test statistic is calculated according to Equation (16).

(16) $$Q=\frac{12}{nk\left(k+1\right)}\sum_{j=1}^{k}R_{j}^{2}-3n\left(k+1\right),$$

where n is the number of blocks, k is the number of groups, and $R_{j}$ is the rank sum for j-th group. When n and k are large, Q follows approximately a $\chi^{2}$ distribution with k−1 degrees of freedom.

From the Friedman test statistical results in Table 6, EH-NGO consistently secured the top positions in both mean ranking (M.R) and total ranking (T.R) across the three major test scenarios of CEC2017 (30-dimensional) and CEC2022 (10-dimensional, 20-dimensional), with significant advantages: in the CEC2017 30-dimensional scenario, its mean ranking was only 1.80, far lower than the second-ranked LSHADE-cnEpSin (3.33) and the original NGO (4.73), and the gap with the lowest-ranked NRBO (8.60) was as high as 6.8 ranking units; in the CEC2022 10-dimensional scenario, EH-NGO achieved a mean ranking of 2.08, outperforming algorithms such as LSHADE-SPACMA (4.92) and CPO (3.67), with minimal ranking fluctuations; even in the CEC2022 20-dimensional high-dimensional scenario, its mean ranking remained at 2.00, leading the second-best algorithm LSHADE-SPACMA (3.83) by 1.83 units. Additionally, the Friedman test statistic Q-values for all scenarios exceeded the critical value of 15.51 (for α = 0.05, degrees of freedom = 8), such as Q = 42.37 for the CEC2017 30-dimensional scenario, proving that EH-NGO’s ranking advantage is not random but statistically significant.

**Figure 10:**
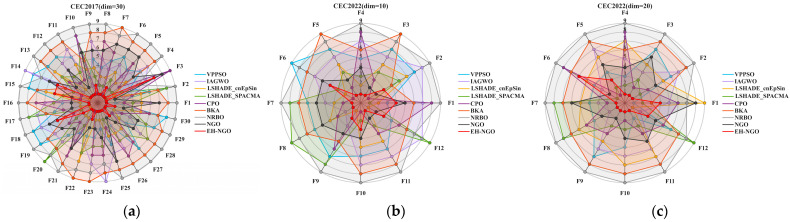
Distribution of rankings of different algorithms.

Further corroborating the algorithm ranking distribution in Figure 10, the scope and stability of EH-NGO’s advantages are reinforced. In the CEC2017 30-dimensional function ranking distribution (Figure 10a), EH-NGO ranked first in 21 functions, including F1 (unimodal), F10 (multimodal), and F22 (high-dimensional complex functions), and ranked 2nd to 3rd in only 9 functions such as F7 and F19, with no function ranking below 3rd, exhibiting a “high concentration at the top” characteristic in its ranking distribution, while the original NGO ranked in the top 3 in only 5 functions such as F5 and F8, and its ranking dropped to 6th–9th in high-dimensional complex functions like F12 and F30, with algorithms like VPPSO and BKA ranking below 5th in over 15 functions. In the CEC2022 10-dimensional scenario (Figure 10b), EH-NGO maintained the top ranking in 10 functions such as F3, F9, and F11, and ranked 2nd in the remaining 2 functions, showing no significant performance weaknesses; in comparison, although LSHADE-cnEpSin ranked first in 3 functions such as F2 and F6, its ranking dropped to 4th–5th in functions like F5 and F10, demonstrating far inferior stability to EH-NGO. The CEC2022 20-dimensional scenario (Figure 10c) shows that EH-NGO ranked first in 8 functions such as F2, F4, and F10; 2nd in 4 functions like F7 and F12; and maintained 3rd place even in F11 (high-dimensional multi-extremum function), while algorithms like NRBO and BKA ranked below 7th in most functions, with some even ranking 9th, highlighting severe performance degradation issues in high-dimensional scenarios.

## 4. EH-NGO for Feature Selection

In the era of big data, the availability of massive datasets has brought significant benefits to machine learning models. However, the sheer scale and complexity of these datasets also introduce the challenge of overfitting. Overfitting occurs when a model achieves excellent performance on the training set but performs poorly on the test set, mainly due to the complexity of the dataset and the lack of representative features that are beneficial to the model [45,46]. Feature Selection (FS) addresses this issue by reducing data dimensionality, thereby enhancing the model’s generalization ability and classification accuracy. The objective of FS is to identify and retain the most representative and informative features. Swarm Intelligence (SI) has demonstrated remarkable capability in finding optimal solutions, efficiently navigating vast search spaces to locate global optima. Its optimization power makes it a powerful tool for tackling FS problems [47,48]. In this section, the proposed EH-NGO is applied to feature selection tasks to validate its effectiveness in these scenarios.

### 4.1. The Proposed EH-NGO-KNN

The feature selection (FS) problem aims to extract a subset from a larger feature set to achieve specific optimization objectives, such as improving model performance or reducing computational complexity. Its mathematical formulation varies depending on the objectives and constraints, and the formulation adopted in this study is presented below.

K-Nearest Neighbor (KNN) is a widely used machine learning classifier with applications across multiple domains, including stock prediction, disease diagnosis, and casting process parameter optimization. KNN classifies samples based on Euclidean distance, which is mathematically expressed as [49,50]

(17) $$Dis(x_{1}-x_{2})=\sqrt{\sum_{k=1}^{N}{\left(x_{1k}-x_{2k}\right)}^{2}},$$

**Figure 11:**
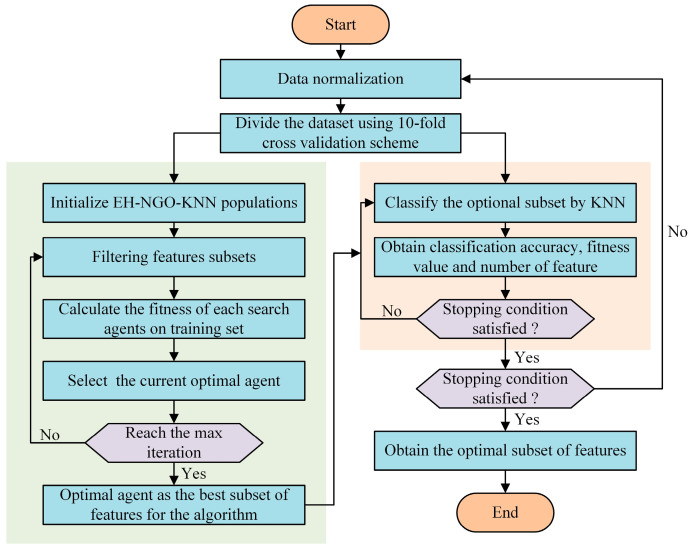
Flowchart of EH-NGO-KNN.

The core objective of FS is to reduce the number of features in a dataset by selecting representative features that can improve the performance of the method—an optimization goal that aligns well with Swarm Intelligence (SI). Specifically, the fewer the selected features and the higher the classification or prediction accuracy, the better the optimization outcome of the algorithm. In this study, the KNN classifier is employed to evaluate the selected features and their classification accuracy. To this end, a method named EH-NGO-KNN is proposed for feature selection, and its workflow is illustrated in Figure 11. Furthermore, assume the dataset contains D features: $X=\left\{x_{1},x_{2},\cdots ,x_{N}\right\}$, where $x_{i}$ is a D-dimensional feature vector, and y is the response variable. The objective is to select a subset from the original D features to maximize or minimize a specific objective function. A fitness function that integrates both the number of features and classification accuracy is defined to evaluate all methods, expressed as

(18) $$\mathrm{Minimize}:\;fitness=\alpha \times CER+(1-\alpha )\times \frac{\left|R\right|}{\left|D\right|},$$

where CER denotes the classification error rate (a core metric for quantifying the proportion of misclassified samples in a classification task), calculated by Equation (18); α is a random number sampled from a uniform distribution in the interval [0, 1], serving as a weight regulator to balance the importance of CER and feature selection ratio in the fitness function); |R| represents the number of selected features, and |D| is the total number of features.

(19) $$Accuracy=\frac{TP+TN}{TP+TN+FP+FN},$$

where TP denotes the number of positive samples correctly identified, TN represents the number of negative samples correctly identified, FP indicates negative samples misclassified as positive, and FN refers to positive samples misclassified as negative. The optimization problem is subject to the following constraint:

(20) $$\mathrm{Subject}\;\mathrm{to}:\;\sum_{j=1}^{D}x_{i,j}\leq K,i\in \{1,\ldots ,N\},$$

where K denotes the maximum number of features allowed to be selected (if such a constraint is imposed). The decision variables are defined as

(21) $$\mathrm{With}:\;x_{i,j}\in \{0,1\},i\in \{1,\ldots ,N\},j\in \{1,\ldots ,D\},$$

where $x_{i,j}$ is a binary decision variable indicating whether feature j is selected for sample i: if $x_{i,j}=1$, the feature is selected; otherwise, if $x_{i,j}=0$, the feature is not selected.

### 4.2. Experimental Study and Discussion

In the feature selection experiments, 22 publicly available datasets were employed to evaluate the performance of EH-NGO-KNN. It is worth noting that these datasets vary in complexity. Based on the number of features, they were categorized into three groups: small datasets (fewer than 20 features), medium datasets (21–100 features), and large datasets (more than 100 features). Each dataset was partitioned into training, testing, and validation subsets using cross-validation, after which the KNN classifier (with the number of neighbors set to 5) was applied to compute the objective function defined in Equation (18). Detailed information on the datasets is provided in Table 7.

Furthermore, EH-NGO-KNN was benchmarked against several recently proposed algorithms to assess its effectiveness. To ensure fairness and eliminate randomness, the population size was fixed at 30, the maximum number of iterations was set to 100, and each algorithm was independently executed 30 times. The experimental results were statistically summarized, with the best outcomes highlighted in bold.

All experiments were conducted in a computing environment consisting of the Windows 11 operating system, an AMD Ryzen 7 9700X 8-Core Processor (3.80 GHz), 48 GB RAM, and the MATLAB 2024b software platform.

**Figure 12:**
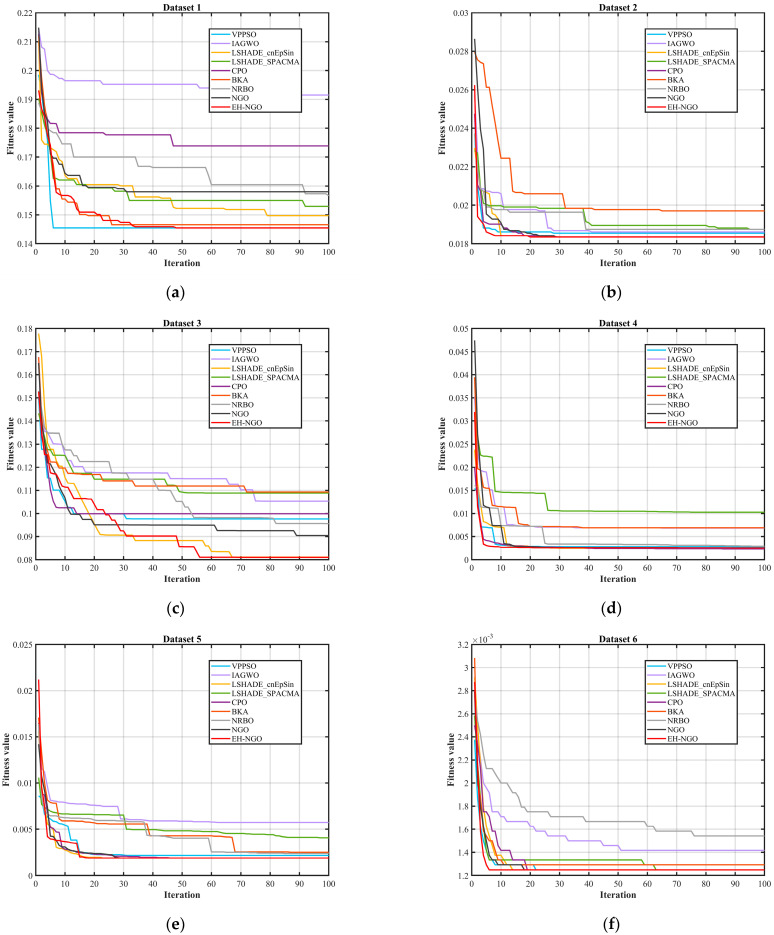

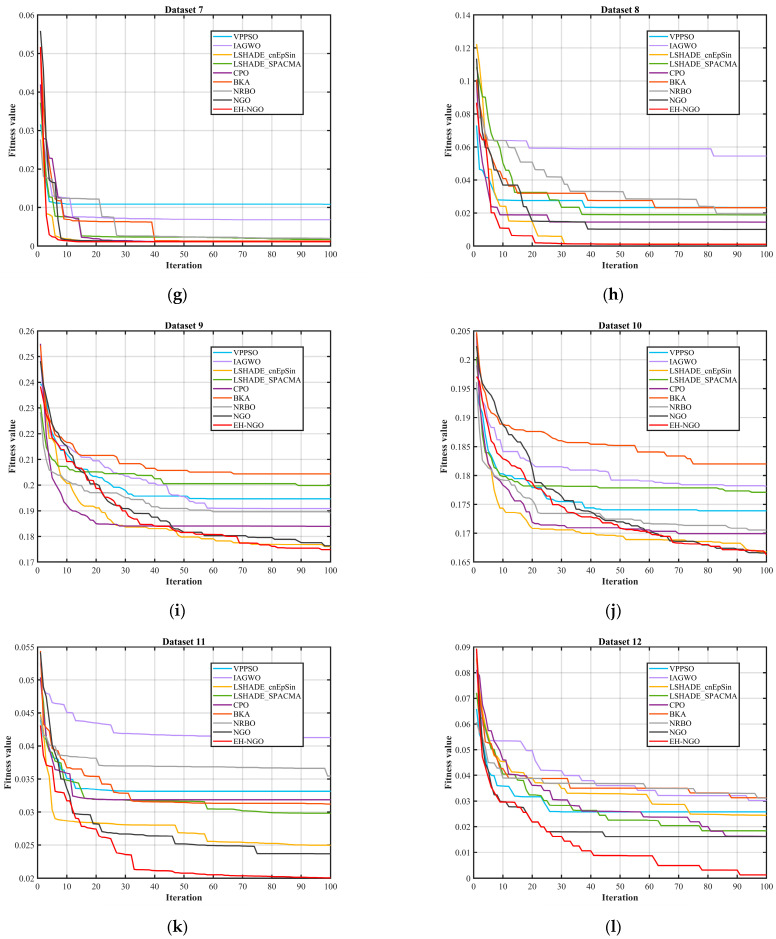

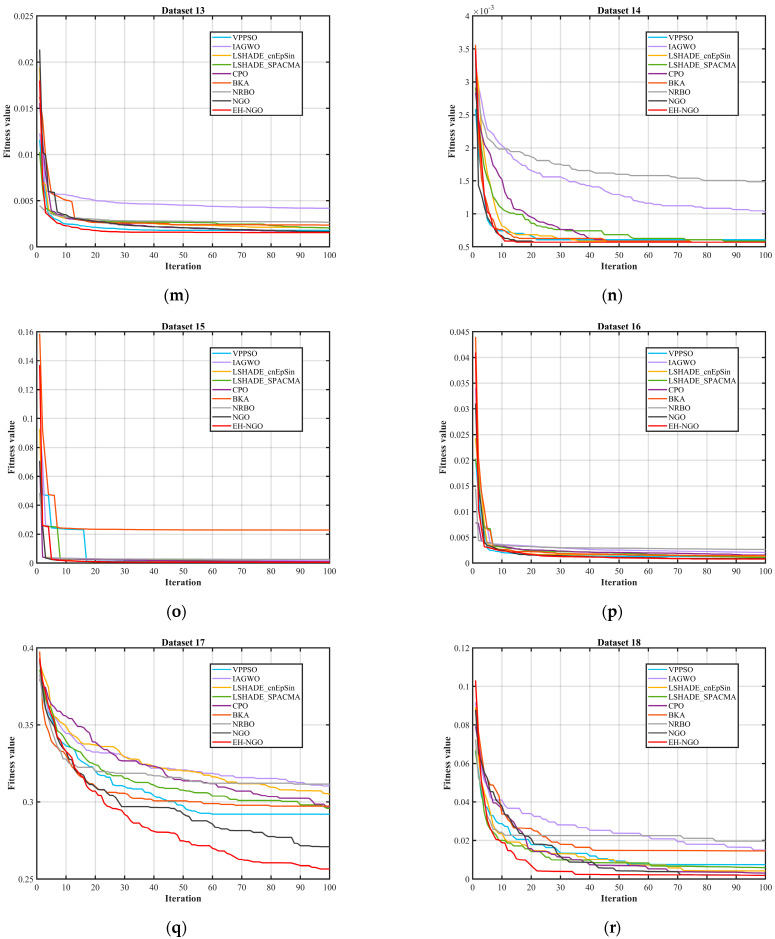

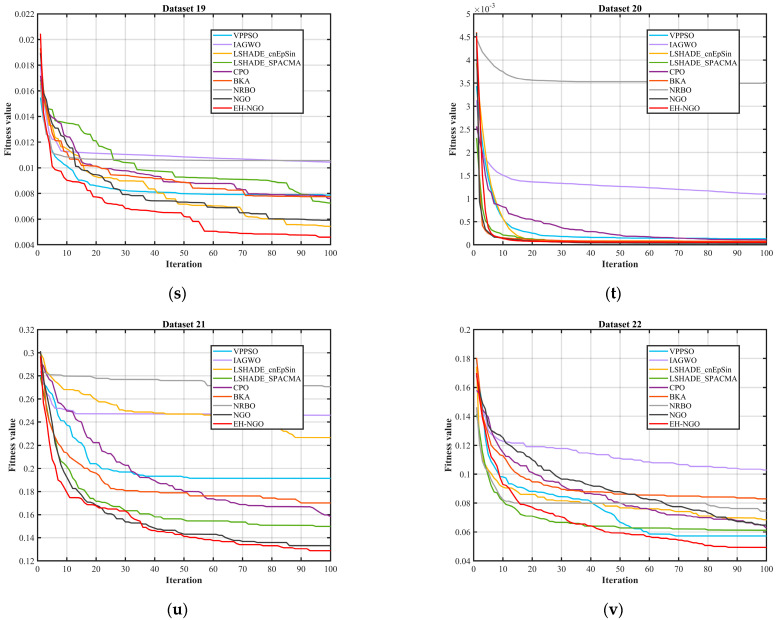
Comparison of the convergence speed of different algorithms.

This study adopts four metrics to evaluate the performance of the proposed algorithm and its competitors: the average classification accuracy, the mean and standard deviation of fitness values, and the number of selected features. These indicators are used to assess the robustness of all compared methods. When candidate solutions are widely distributed across the search space, the probability of locating the optimal solution increases significantly. In addition, the average ranking test is employed to evaluate the overall performance of EH-NGO and other methods, with the best results under different metrics highlighted in bold. Figure 12 and Table 7, Table 8 and Table 9 comprehensively validate the effectiveness of the EH-NGO-based feature selection method (EH-NGO-KNN) across 18 datasets of varying scales (small, medium, and large, with feature counts ranging from 9 to 166) from four perspectives: convergence efficiency, fitness performance, classification accuracy, and feature selection capability. For all experiments, the population size was set to 30, the maximum number of iterations was fixed at 100, and the KNN classifier (neighbors = 5) was adopted for performance evaluation, while EH-NGO-KNN was compared with eight algorithms, including VPPSO and IAGWO.

By combining the algorithm convergence curve in Figure 12 and the quantitative experimental data in Table 8, Table 9 and Table 10, the feature selection performance of EH-NGO-KNN can be comprehensively evaluated across 22 datasets of varying sizes (with 9 to 166 features). All analyses are based on the experimental results from this text. From the convergence curve in Figure 12, EH-NGO demonstrates faster convergence speed and superior convergence accuracy across all datasets: On the low-dimensional Dataset 2 (Breast Cancer Wisconsin, 10 features), EH-NGO requires only 30 iterations to reach a stable fitness value (approximately 6.4 × 10^−2^), whereas the original NGO requires over 60 iterations, and algorithms such as VPPSO and IAGWO struggle to surpass the 8 × 10^−2^ fitness range. On medium- to high-dimensional datasets such as Dataset 12 (Ionosphere, 34 features) and Dataset 18 (Musk Version 1, 166 features), EH-NGO’s convergence curve consistently lies below all comparative algorithms. After 100 iterations, its fitness value is about 30–40% lower than that of the original NGO, with no significant fluctuations, reflecting its ability to quickly focus on core feature subsets during iterative optimization for feature selection and avoid convergence stagnation caused by local optima.

The fitness results in Table 8 further quantify the optimization effect of EH-NGO: Across the 18 datasets, EH-NGO achieves the lowest or second-lowest average fitness values, with significantly smaller standard deviations. For example, in Dataset 5 (Congressional Voting Records, 16 features), EH-NGO’s average fitness value is 2.6210 × 10^−3^ with a standard deviation of only 7.8573 × 10^−3^, far lower than VPPSO’s 5.3744 × 10^−3^ (Std = 1.0077 × 10^−3^) and IAGWO’s 9.4231 × 10^−3^ (Std = 8.4453 × 10^−3^). In the high-dimensional Dataset 18, EH-NGO’s average fitness value is 9.3409 × 10^−3^ with a standard deviation of 2.4236 × 10^−4^, approximately 47.6% lower than BKA’s 1.7829 × 10^−3^ (Std = 4.1523 × 10^−3^). This demonstrates its advantage in balancing “classification error rate” and “feature subset size,” with stronger stability in optimization results.

The classification accuracy data in Table 9 confirms the effectiveness of the features selected by EH-NGO: Among the 18 datasets, EH-NGO-KNN achieves 100% classification accuracy on 13 datasets. For instance, on Dataset 15 (Lung Cancer, 56 features), the accuracy reaches 71.65%, which is 1.57% and 4.52% higher than the original NGO’s 70.08% and VPPSO’s 67.13%, respectively. Even on Dataset 14 (Soybean, 35 features, 47 samples), which has low samples and high dimensionality, EH-NGO maintains 100% accuracy, while algorithms like NRBO and BKA achieve only around 99.50% accuracy. This indicates that the feature subsets selected by EH-NGO accurately retain discriminant information from the data, effectively supporting the performance of the KNN classifier.

The feature selection quantity results in Table 10 highlight EH-NGO’s dimensionality reduction capability: Across the 22 datasets, EH-NGO selects an average of only 5.66 features, which is 44.2% fewer than IAGWO’s 10.15 and 54.0% fewer than NRBO’s 12.30. The advantage is more pronounced in high-dimensional scenarios. For example, in Dataset 20 (Malware Executable Detection, 531 features), EH-NGO selects only 13.50 features, far fewer than the original NGO’s 35.57 and IAGWO’s 182.30. In Dataset 18 (166 features), EH-NGO selects only 60.53 features while maintaining 100% accuracy, approximately 20% fewer than VPPSO’s 76.37 features. This fully demonstrates its ability to eliminate redundancy and focus on core features.

Integrating the convergence trend in Figure 12 and the quantitative metrics in Table 8, Table 9 and Table 10, it is evident that within the experimental framework of this article, EH-NGO performs exceptionally well in feature selection tasks of varying scales, owing to its faster convergence speed, superior fitness values, higher classification accuracy, and stronger dimensionality reduction capability. The strategies designed for EH-NGO, such as elite guidance and vertical crossover mutation, effectively address the issues of traditional algorithms, such as “slow convergence, susceptibility to overfitting, and incomplete dimensionality reduction,” validating the practicality and superiority of EH-NGO-KNN in the field of feature selection.

## 5. Summary and Prospect

This study addresses the limitations of traditional metaheuristic algorithms in feature selection, such as blind search, premature convergence, and inefficient boundary handling, by proposing an improved algorithm based on Northern Goshawk Optimization (NGO), namely the Elite-guided Hybrid Northern Goshawk Optimization (EH-NGO). The proposed algorithm integrates three complementary strategies to achieve performance breakthroughs. The elite-guided search strategy constructs an “elite pool” consisting of the top five individuals and employs a dual-selection mechanism to guide population evolution, thereby improving convergence directionality. The vertical crossover mutation strategy generates new solutions through linear combinations across individual dimensions, which enhances population diversity and strengthens the ability to escape local optima. Meanwhile, the global best-guided boundary control strategy transforms the traditional passive penalty-based boundary handling into an active guidance process, effectively reducing ineffective searches.

Experimental validation on 30 benchmark functions from the CEC2017 test suite shows that EH-NGO significantly outperforms eight state-of-the-art algorithms, including VPPSO and IAGWO, achieving superior global optimization performance with an average Friedman rank of only 1.80, 2.08 and 2.0, ranking first overall. When integrated with the KNN classifier, the resulting EH-NGO-KNN feature selection method demonstrates excellent classification capability on 22 datasets of varying scales (with feature sizes ranging from 9 to 166). Specifically, it achieves 100% classification accuracy on 13 datasets, selects an average of only 5.66 features (representing a 44.2% reduction compared with IAGWO), and maintains fast convergence and strong stability. These results verify the synergistic advantages of EH-NGO in achieving high accuracy, reduced feature dimensionality, and accelerated convergence.

Looking ahead, future work may further enhance EH-NGO by incorporating reinforcement learning to enable dynamic adaptation of strategy parameters, extending it to multi-objective optimization to balance classification accuracy, feature subset size, and computational efficiency, and integrating it with deep learning models to improve its capability in handling ultra-large datasets. In terms of applications, the algorithm can be extended to fields such as computer vision and bioinformatics, where it may support multimodal feature selection and incremental optimization in dynamic feature stream scenarios. From an engineering perspective, the development of parallel-accelerated implementations and open-source toolkits in C++ or Python, combined with validation in practical domains such as healthcare and finance, will facilitate the transition of EH-NGO from theoretical research to industrial applications, ultimately providing a more efficient solution for complex optimization problems and high-dimensional data analysis.

## Figures and Tables

**Table 1:** Comparison Table of improvements between the original NGO and EH-NGO algorithms.

Comparison Dimension	Original NGO Algorithm	EH-NGO Algorithm
Search Guidance Mechanism (Exploration Phase)	Relies entirely on randomly selected individuals for position updates, resulting in blind search directions and failure to utilize high-quality solution information within the population	Constructs an “elite pool” containing the top 5 fittest individuals and adopts a dual-selection mechanism: 10% probability of using the average position of elites and 90% probability of randomly selecting a single elite as the guiding individual. The position update formula is reconstructed (Equation (8))
Population Diversity Maintenance (Exploitation Phase)	Generates perturbations only through random scaling of an individual’s own position; the mechanism is simple and fails to break the similarity among individuals	Introduces a vertical crossover mutation strategy: randomly selects two different dimensions $\left(j_{1}\neq j_{2}\right)$ of an individual, generates new dimension values through linear combination (Equation (9)), and keeps the remaining dimensions unchanged
Boundary Handling Strategy	Adopts a “penalty-based” passive handling method: directly resets out-of-bound individuals to the boundary, interrupting the natural evolutionary trajectory of solutions and wasting search direction information	Implements an active boundary control guided by the global best solution: when an individual exceeds the boundary, it is guided toward high-quality regions based on the current global best solution (X_best) (Equation (10)). For example, if X(i,j)>ub(j), the individual is guided to the vicinity of X_best(j)

**Table 2:** Compare algorithm parameter settings.

Algorithms	Name of the Parameter	Value of the Parameter
VPPSO	$c_{1},\;c_{2},\;w,\;\alpha ,\;N_{1},\;N_{2}$	2, 2, 0.8, [1, 0], 0.15, 0.15
IAGWO	$v_{rand}\left(t\right),\;a,\;\omega ,\;\theta$	[−20, 20], [0, 2], [0.3, 0.9], 0.5
LSHADE_cnEpSin	$p,\;\left|A\right|,\;H$	0.11, 1.4, 5
LSHADE_SPACMA	$p,\;H,\;Arc_{rate},\;F_{CP},\;c$	0.11, 1.4, 5, 0.5, 0.5
CPO	$\alpha ,\;N_{min},\;T_{f},\;T$	0.1,80,0.5,2
BKA	P, r	0.9, [0, 1]
NRBO	DF	0.6
NGO	I, R	$\left\{1,\;2\right\},\;0.02$
EH-NGO	I, R, α	$\left\{1,\;2\right\},\;\left[0,\;1\right],\;\left[0,\;1\right]$

**Table 3:** Experimental results of CEC2017 (dim = 30).

Function	Metric	VPPSO	IAGWO	LSHADE_cnEpSin	LSHADE_SPACMA	CPO	BKA	NRBO	NGO	EH-NGO
F1	Ave	6.7701 × 10^6^	1.3588 × 10^6^	2.3024 × 10^6^	5.8890 × 10^6^	7.5912 × 10^5^	1.0482 × 10^10^	1.7668 × 10^10^	7.1775 × 10^7^	**1.0221 × 10^4^**
	Std	1.5034 × 10^7^	4.7422 × 10^5^	2.5187 × 10^6^	1.3329 × 10^7^	6.7768 × 10^5^	1.0633 × 10^10^	4.1308 × 10^9^	2.4677 × 10^8^	**1.0226 × 10^4^**
	Rank	2	6	5	4	3	8	9	7	**1**
F2	Ave	7.1612 × 10^23^	9.0249 × 10^34^	9.0962 × 10^24^	1.0000 × 10^30^	1.5137 × 10^20^	1.4392 × 10^41^	8.0804 × 10^37^	3.8782 × 10^18^	**1.0837 × 10^15^**
	Std	2.4369 × 10^24^	4.0792 × 10^35^	4.8614 × 10^25^	**1.4314 × 10^14^**	3.5602 × 10^20^	7.7247 × 10^41^	4.4116 × 10^38^	9.7321 × 10^18^	5.2406 × 10^15^
	Rank	6	3	5	8	4	7	9	2	**1**
F3	Ave	5.1982 × 10^4^	5.8349 × 10^4^	4.1759 × 10^4^	5.1159 × 10^4^	6.5313 × 10^4^	**3.5277 × 10^4^**	5.3722 × 10^4^	6.3074 × 10^4^	5.9325 × 10^4^
	Std	1.2211 × 10^4^	1.7822 × 10^4^	2.3208 × 10^4^	4.7146 × 10^4^	1.2689 × 10^4^	1.5020 × 10^4^	**8.6874 × 10^3^**	9.5068 × 10^3^	1.1659 × 10^4^
	Rank	4	6	2	3	9	**1**	5	8	7
F4	Ave	5.3422 × 10^2^	6.6528 × 10^2^	5.2201 × 10^2^	5.1752 × 10^2^	5.2270 × 10^2^	2.0741 × 10^3^	1.9708 × 10^3^	5.2892 × 10^2^	**4.8646 × 10^2^**
	Std	2.8693 × 10^1^	3.0350 × 10^2^	2.5404 × 10^1^	3.3106 × 10^1^	1.8822 × 10^1^	3.0674 × 10^3^	1.2020 × 10^3^	**1.8392 × 10^1^**	2.7298 × 10^1^
	Rank	6	7	4	2	3	8	9	5	**1**
F5	Ave	6.5513 × 10^2^	6.4721 × 10^2^	6.0678 × 10^2^	6.0645 × 10^2^	6.9601 × 10^2^	7.4140 × 10^2^	8.4421 × 10^2^	6.9780 × 10^2^	**5.8466 × 10^2^**
	Std	3.8292 × 10^1^	2.8587 × 10^1^	2.7864 × 10^1^	2.5143 × 10^1^	1.7476 × 10^1^	4.9016 × 10^1^	3.3122 × 10^1^	2.8719 × 10^1^	**1.5956 × 10^1^**
	Rank	5	4	3	2	6	8	9	7	**1**
F6	Ave	6.3678 × 10^2^	6.2284 × 10^2^	6.0922 × 10^2^	6.0800 × 10^2^	**6.0193 × 10^2^**	6.6050 × 10^2^	6.7200 × 10^2^	6.4190 × 10^2^	6.0435 × 10^2^
	Std	9.3757	7.0311	4.0264	4.5052	**7.5198 × 10^−1^**	7.4914	1.0209 × 10^1^	9.0750	1.6391
	Rank	6	5	4	3	**1**	8	9	7	2
F7	Ave	9.2783 × 10^2^	9.0957 × 10^2^	8.8064 × 10^2^	8.7346 × 10^2^	9.4425 × 10^2^	1.2319 × 10^3^	1.2254 × 10^3^	1.0420 × 10^3^	**8.4076 × 10^2^**
	Std	7.2504 × 10^1^	5.5547 × 10^1^	3.7960 × 10^1^	4.4729 × 10^1^	1.8698 × 10^1^	8.0295 × 10^1^	7.3450 × 10^1^	5.0651 × 10^1^	**1.7791 × 10^1^**
	Rank	5	4	3	2	6	9	8	7	**1**
F8	Ave	9.2348 × 10^2^	9.1862 × 10^2^	8.9182 × 10^2^	8.9136 × 10^2^	9.8448 × 10^2^	9.9063 × 10^2^	1.0833 × 10^3^	9.6080 × 10^2^	**8.8278 × 10^2^**
	Std	4.0865 × 10^1^	1.8405 × 10^1^	1.8522 × 10^1^	1.9218 × 10^1^	1.7027 × 10^1^	4.9145 × 10^1^	2.9866 × 10^1^	2.5938 × 10^1^	**1.6058 × 10^1^**
	Rank	4	5	3	2	7	8	9	6	**1**
F9	Ave	3.2201 × 10^3^	4.5574 × 10^3^	2.1162 × 10^3^	2.1068 × 10^3^	**1.4249 × 10^3^**	5.0929 × 10^3^	6.7836 × 10^3^	3.8156 × 10^3^	1.4901 × 10^3^
	Std	5.0150 × 10^2^	1.1129 × 10^3^	6.6860 × 10^2^	8.4398 × 10^2^	4.1110 × 10^2^	6.6213 × 10^2^	1.6703 × 10^3^	6.8746 × 10^2^	**3.0161 × 10^2^**
	Rank	5	7	4	3	**1**	8	9	6	2
F10	Ave	5.0775 × 10^3^	4.4519 × 10^3^	5.2057 × 10^3^	6.1198 × 10^3^	7.5335 × 10^3^	5.5374 × 10^3^	7.8506 × 10^3^	5.5785 × 10^3^	**3.9251 × 10^3^**
	Std	7.0916 × 10^2^	6.7079 × 10^2^	8.1014 × 10^2^	1.2573 × 10^3^	3.8928 × 10^2^	1.1498 × 10^3^	6.6938 × 10^2^	4.0279 × 10^2^	**3.4327 × 10^2^**
	Rank	3	2	4	7	8	5	9	6	**1**
F11	Ave	1.4462 × 10^3^	2.0784 × 10^3^	1.3239 × 10^3^	1.3072 × 10^3^	1.2747 × 10^3^	1.9566 × 10^3^	2.8481 × 10^3^	1.2439 × 10^3^	**1.1834 × 10^3^**
	Std	1.3115 × 10^2^	1.5024 × 10^3^	5.8516 × 10^1^	7.0855 × 10^1^	2.9038 × 10^1^	1.8072 × 10^3^	9.0661 × 10^2^	3.7634 × 10^1^	**2.8092 × 10^1^**
	Rank	6	7	5	4	3	8	9	2	**1**
F12	Ave	3.5443 × 10^7^	2.4040 × 10^7^	1.3839 × 10^6^	1.8928 × 10^6^	1.1637 × 10^6^	5.2511 × 10^8^	1.4814 × 10^9^	1.8359 × 10^6^	**5.0108 × 10^5^**
	Std	4.8444 × 10^7^	4.2985 × 10^7^	1.3691 × 10^6^	1.7671 × 10^6^	6.1051 × 10^5^	1.6860 × 10^9^	6.1991 × 10^8^	7.0786 × 10^5^	**4.8291 × 10^5^**
	Rank	7	6	2	4	3	8	9	5	**1**
F13	Ave	9.8623 × 10^4^	1.8356 × 10^4^	2.8724 × 10^4^	3.0371 × 10^4^	2.0486 × 10^4^	9.4285 × 10^7^	3.7397 × 10^8^	1.9094 × 10^4^	**1.4466 × 10^4^**
	Std	3.8084 × 10^4^	1.8546 × 10^4^	1.8284 × 10^4^	2.2182 × 10^4^	**1.4325 × 10^4^**	2.7053 × 10^8^	2.8572 × 10^8^	1.5117 × 10^4^	1.5339 × 10^4^
	Rank	7	2	6	5	4	8	9	3	**1**
F14	Ave	1.3409 × 10^5^	7.4229 × 10^5^	3.4056 × 10^3^	5.9625 × 10^4^	**2.0472 × 10^3^**	6.0051 × 10^4^	1.7176 × 10^5^	1.9570 × 10^4^	7.0856 × 10^3^
	Std	1.6004 × 10^5^	6.1363 × 10^5^	8.3699 × 10^3^	3.0830 × 10^5^	**5.4285 × 10^2^**	2.8293 × 10^5^	2.6770 × 10^5^	2.2080 × 10^4^	4.9534 × 10^3^
	Rank	8	9	**1**	3	2	4	7	6	5
F15	Ave	9.7067 × 10^4^	5.2368 × 10^3^	1.2765 × 10^4^	7.3739 × 10^3^	**4.2960 × 10^3^**	5.0369 × 10^4^	2.6347 × 10^6^	6.9114 × 10^3^	1.1713 × 10^4^
	Std	3.1231 × 10^5^	4.1782 × 10^3^	1.1021 × 10^4^	6.4436 × 10^3^	**1.7240 × 10^3^**	6.1700 × 10^4^	3.8989 × 10^6^	6.1272 × 10^3^	1.0751 × 10^4^
	Rank	8	**1**	6	4	2	7	9	3	5
F16	Ave	2.8709 × 10^3^	3.0727 × 10^3^	2.4760 × 10^3^	3.0755 × 10^3^	3.0673 × 10^3^	3.2328 × 10^3^	3.7986 × 10^3^	2.7863 × 10^3^	**2.2830 × 10^3^**
	Std	3.0005 × 10^2^	3.9024 × 10^2^	2.1676 × 10^2^	6.9928 × 10^2^	2.3462 × 10^2^	4.4256 × 10^2^	3.6424 × 10^2^	1.9464 × 10^2^	**1.8638 × 10^2^**
	Rank	4	6	2	5	7	8	9	3	**1**
F17	Ave	2.2066 × 10^3^	2.3804 × 10^3^	1.9875 × 10^3^	2.7183 × 10^3^	2.0870 × 10^3^	2.3427 × 10^3^	2.7047 × 10^3^	2.0179 × 10^3^	**1.8991 × 10^3^**
	Std	1.9833 × 10^2^	2.4614 × 10^2^	1.0717 × 10^2^	5.2303 × 10^2^	1.2306 × 10^2^	2.6038 × 10^2^	2.6711 × 10^2^	1.1290 × 10^2^	**9.7814 × 10^1^**
	Rank	5	7	2	8	4	6	9	3	**1**
F18	Ave	1.6169 × 10^6^	9.5230 × 10^5^	**5.8067 × 10^4^**	2.4660 × 10^5^	1.3528 × 10^5^	6.7391 × 10^5^	1.8571 × 10^6^	3.6769 × 10^5^	2.3107 × 10^5^
	Std	1.5848 × 10^6^	1.2662 × 10^6^	**2.8314 × 10^4^**	1.0203 × 10^6^	9.7300 × 10^4^	2.4922 × 10^6^	1.9427 × 10^6^	3.7447 × 10^5^	1.4560 × 10^5^
	Rank	8	7	**1**	2	3	4	9	6	5
F19	Ave	2.0910 × 10^6^	8.0228 × 10^3^	**3.9960 × 10^3^**	6.6174 × 10^3^	5.2405 × 10^3^	2.7594 × 10^6^	2.1833 × 10^7^	7.0696 × 10^3^	7.1663 × 10^3^
	Std	1.3243 × 10^6^	6.9079 × 10^3^	**2.5909 × 10^3^**	1.0138 × 10^4^	3.3125 × 10^3^	1.3786 × 10^7^	2.5739 × 10^7^	6.3292 × 10^3^	7.3444 × 10^3^
	Rank	8	6	**1**	3	4	7	9	5	2
F20	Ave	2.5628 × 10^3^	2.5809 × 10^3^	2.3691 × 10^3^	2.9736 × 10^3^	2.4786 × 10^3^	2.5903 × 10^3^	2.7288 × 10^3^	2.4608 × 10^3^	**2.2526 × 10^3^**
	Std	1.5083 × 10^2^	2.2717 × 10^2^	1.0894 × 10^2^	2.5517 × 10^2^	1.3515 × 10^2^	1.7415 × 10^2^	1.8340 × 10^2^	1.1011 × 10^2^	**1.0260 × 10^2^**
	Rank	5	6	2	9	3	7	8	4	**1**
F21	Ave	2.4277 × 10^3^	2.4543 × 10^3^	2.3925 × 10^3^	2.4039 × 10^3^	2.4839 × 10^3^	2.5599 × 10^3^	2.6053 × 10^3^	2.4666 × 10^3^	**2.3855 × 10^3^**
	Std	2.9707 × 10^1^	3.1918 × 10^1^	1.9622 × 10^1^	2.3233 × 10^1^	1.9181 × 10^1^	6.5696 × 10^1^	4.5107 × 10^1^	2.7632 × 10^1^	**1.6568 × 10^1^**
	Rank	4	5	2	3	7	8	9	6	**1**
F22	Ave	3.2640 × 10^3^	3.9169 × 10^3^	4.2463 × 10^3^	3.2406 × 10^3^	**2.3087 × 10^3^**	6.7684 × 10^3^	5.9493 × 10^3^	2.3279 × 10^3^	3.6220 × 10^3^
	Std	1.7157 × 10^3^	2.1794 × 10^3^	2.3824 × 10^3^	2.0322 × 10^3^	**3.4447**	1.5614 × 10^3^	2.2365 × 10^3^	1.8268 × 10^1^	1.6550 × 10^3^
	Rank	4	5	7	6	**1**	9	8	3	2
F23	Ave	2.8123 × 10^3^	3.0567 × 10^3^	2.7679 × 10^3^	2.8261 × 10^3^	2.8466 × 10^3^	3.1261 × 10^3^	3.0742 × 10^3^	2.8171 × 10^3^	**2.7484 × 10^3^**
	Std	4.5975 × 10^1^	1.4697 × 10^2^	2.9552 × 10^1^	7.0806 × 10^1^	1.6766 × 10^1^	1.2245 × 10^2^	6.2743 × 10^1^	3.3866 × 10^1^	**1.6707 × 10^1^**
	Rank	4	7	2	5	6	9	8	3	**1**
F24	Ave	2.9521 × 10^3^	3.3411 × 10^3^	2.9414 × 10^3^	3.0212 × 10^3^	3.0222 × 10^3^	3.3298 × 10^3^	3.1975 × 10^3^	2.9747 × 10^3^	**2.9309 × 10^3^**
	Std	3.8149 × 10^1^	1.5393 × 10^2^	2.7832 × 10^1^	9.2582 × 10^1^	**1.7769 × 10^1^**	1.4446 × 10^2^	7.4267 × 10^1^	2.7987 × 10^1^	3.2615 × 10^1^
	Rank	3	9	2	5	6	8	7	4	**1**
F25	Ave	2.9435 × 10^3^	2.9478 × 10^3^	2.9207 × 10^3^	2.9090 × 10^3^	2.9110 × 10^3^	3.1521 × 10^3^	3.3891 × 10^3^	2.9560 × 10^3^	**2.8906 × 10^3^**
	Std	2.5593 × 10^1^	2.9472 × 10^1^	2.1242 × 10^1^	2.0096 × 10^1^	2.1091 × 10^1^	2.3953 × 10^2^	1.7145 × 10^2^	3.0878 × 10^1^	**1.0819 × 10^1^**
	Rank	5	6	4	2	3	8	9	7	**1**
F26	Ave	4.6548 × 10^3^	5.0596 × 10^3^	4.7688 × 10^3^	4.7639 × 10^3^	5.0595 × 10^3^	7.9579 × 10^3^	7.6965 × 10^3^	4.4894 × 10^3^	**4.3905 × 10^3^**
	Std	1.1657 × 10^3^	1.6863 × 10^3^	**6.2882 × 10^2^**	1.1732 × 10^3^	1.1815 × 10^3^	1.6700 × 10^3^	9.1003 × 10^2^	1.5084 × 10^3^	6.4992 × 10^2^
	Rank	3	6	4	5	7	8	9	2	**1**
F27	Ave	3.3151 × 10^3^	3.2356 × 10^3^	3.2546 × 10^3^	3.5930 × 10^3^	3.2751 × 10^3^	3.3853 × 10^3^	3.4926 × 10^3^	3.2424 × 10^3^	**3.2124 × 10^3^**
	Std	8.8072 × 10^1^	1.2851 × 10^2^	2.8384 × 10^1^	7.7045 × 10^2^	**9.9520**	1.0599 × 10^2^	1.4998 × 10^2^	2.1475 × 10^1^	1.3562 × 10^1^
	Rank	7	**1**	4	5	6	8	9	3	2
F28	Ave	3.3135 × 10^3^	3.3552 × 10^3^	3.2929 × 10^3^	6.9556 × 10^3^	3.2749 × 10^3^	4.2122 × 10^3^	4.0549 × 10^3^	3.3248 × 10^3^	**3.2147 × 10^3^**
	Std	2.9198 × 10^1^	9.0683 × 10^1^	4.3343 × 10^1^	3.9284 × 10^3^	2.9318 × 10^1^	1.0183 × 10^3^	3.3074 × 10^2^	2.9576 × 10^1^	**1.7973 × 10^1^**
	Rank	4	6	3	7	2	8	9	5	**1**
F29	Ave	4.3094 × 10^3^	3.9976 × 10^3^	3.8417 × 10^3^	4.4612 × 10^3^	3.9427 × 10^3^	4.6679 × 10^3^	5.0657 × 10^3^	4.1458 × 10^3^	**3.6102 × 10^3^**
	Std	2.8084 × 10^2^	2.9163 × 10^2^	1.7819 × 10^2^	7.4774 × 10^2^	1.2306 × 10^2^	5.5449 × 10^2^	3.7070 × 10^2^	1.7387 × 10^2^	**8.2394 × 10^1^**
	Rank	7	4	2	6	3	8	9	5	**1**
F30	Ave	8.1356 × 10^6^	4.8415 × 10^6^	4.5788 × 10^4^	6.3536 × 10^4^	1.1463 × 10^5^	7.8434 × 10^6^	9.1763 × 10^7^	2.5393 × 10^4^	**1.1892 × 10^4^**
	Std	6.4836 × 10^6^	2.6487 × 10^7^	3.5294 × 10^4^	8.5202 × 10^4^	6.9202 × 10^4^	2.2779 × 10^7^	6.3466 × 10^7^	1.4150 × 10^4^	**4.8578 × 10^3^**
	Rank	8	**1**	5	4	6	7	9	3	2

**Table 4:** Experimental results of CEC2022(dim = 10).

Function	Metric	VPPSO	IAGWO	LSHADE_cnEpSin	LSHADE_SPACMA	CPO	BKA	NRBO	NGO	EH-NGO
F1	Ave	3.0923 × 10^2^	3.3231 × 10^2^	3.0027 × 10^2^	1.0027 × 10^3^	3.9027 × 10^2^	8.7989 × 10^2^	1.3560 × 10^3^	3.2201 × 10^2^	**3.0000 × 10^2^**
	Std	2.7308 × 10^1^	5.2911 × 10^1^	1.4602	2.7994 × 10^3^	9.2864 × 10^1^	1.5098 × 10^3^	8.8953 × 10^2^	5.0625 × 10^1^	**6.9687 × 10^−3^**
	Rank	3	7	2	**1**	8	6	9	5	4
F2	Ave	4.1010 × 10^2^	4.3692 × 10^2^	4.0718 × 10^2^	4.0580 × 10^2^	**4.0136 × 10^2^**	4.0967 × 10^2^	4.5049 × 10^2^	4.0854 × 10^2^	4.0367 × 10^2^
	Std	1.7392 × 10^1^	3.4769 × 10^1^	1.2271 × 10^1^	3.6830	**2.3778**	2.0235 × 10^1^	2.8106 × 10^1^	2.1267 × 10^1^	3.8422
	Rank	7	8	6	5	**1**	4	9	2	3
F3	Ave	6.0680 × 10^2^	6.0050 × 10^2^	6.0019 × 10^2^	6.0012 × 10^2^	6.0000 × 10^2^	6.2646 × 10^2^	6.2452 × 10^2^	6.0031 × 10^2^	**6.0000 × 10^2^**
	Std	6.1332	8.7898 × 10^−1^	2.6979 × 10^−1^	2.7013 × 10^−1^	**2.2094 × 10^−3^**	8.6621	7.3480	8.6031 × 10^−1^	2.5408 × 10^−3^
	Rank	7	6	5	4	3	9	8	2	**1**
F4	Ave	8.1676 × 10^2^	8.1888 × 10^2^	8.0840 × 10^2^	8.1001 × 10^2^	8.2194 × 10^2^	8.1859 × 10^2^	8.3107 × 10^2^	8.1236 × 10^2^	**8.0832 × 10^2^**
	Std	6.9935	5.8976	**2.7369**	4.5992	6.0607	9.5249	1.0883 × 10^1^	3.4600	3.2894
	Rank	5	7	2	3	8	6	9	4	**1**
F5	Ave	9.1092 × 10^2^	9.2812 × 10^2^	9.0282 × 10^2^	9.0185 × 10^2^	9.0003 × 10^2^	1.1525 × 10^3^	1.0972 × 10^3^	9.0807 × 10^2^	**9.0000 × 10^2^**
	Std	1.6572 × 10^1^	4.9513 × 10^1^	8.3665	3.1551	8.6397 × 10^−2^	1.2581 × 10^2^	1.6289 × 10^2^	2.5488 × 10^1^	**6.0114 × 10^−3^**
	Rank	7	6	5	4	2	9	8	3	**1**
F6	Ave	4.0455 × 10^3^	9.2110 × 10^3^	1.8507 × 10^3^	1.8713 × 10^3^	**1.8298 × 10^3^**	3.1459 × 10^3^	3.7282 × 10^3^	1.9960 × 10^3^	2.2680 × 10^3^
	Std	2.1733 × 10^3^	3.7170 × 10^4^	3.5386 × 10^1^	5.5577 × 10^1^	**1.3229 × 10^1^**	1.6513 × 10^3^	1.8139 × 10^3^	1.0512 × 10^2^	7.7274 × 10^2^
	Rank	9	6	2	3	**1**	7	8	5	4
F7	Ave	2.0400 × 10^3^	2.0167 × 10^3^	2.0154 × 10^3^	2.0835 × 10^3^	2.0113 × 10^3^	2.0527 × 10^3^	2.0615 × 10^3^	2.0186 × 10^3^	**2.0047 × 10^3^**
	Std	1.8439 × 10^1^	8.8307	9.3658	6.2051 × 10^1^	**4.1790**	2.3513 × 10^1^	1.8677 × 10^1^	9.0794	4.4268
	Rank	6	4	3	8	2	7	9	5	**1**
F8	Ave	2.2241 × 10^3^	2.2185 × 10^3^	2.2255 × 10^3^	2.2746 × 10^3^	2.2175 × 10^3^	2.2302 × 10^3^	2.2449 × 10^3^	2.2229 × 10^3^	**2.2125 × 10^3^**
	Std	**4.3510**	6.5982	2.9340 × 10^1^	9.3666 × 10^1^	6.3468	2.2119 × 10^1^	3.6217 × 10^1^	5.1081	7.9216
	Rank	6	2	4	9	3	7	8	5	**1**
F9	Ave	2.5383 × 10^3^	2.5411 × 10^3^	**2.5224 × 10^3^**	2.5504 × 10^3^	2.5293 × 10^3^	2.5400 × 10^3^	2.5954 × 10^3^	2.5293 × 10^3^	2.5293 × 10^3^
	Std	2.6750 × 10^1^	5.5799 × 10^1^	7.2782	2.9050 × 10^1^	4.6632 × 10^−3^	4.0307 × 10^1^	5.3415 × 10^1^	9.7504 × 10^−10^	**2.2416 × 10^−11^**
	Rank	7	3	**1**	8	6	5	9	4	2
F10	Ave	2.5388 × 10^3^	2.5699 × 10^3^	2.5444 × 10^3^	2.5374 × 10^3^	2.5269 × 10^3^	2.5750 × 10^3^	2.5978 × 10^3^	**2.5261 × 10^3^**	2.5657 × 10^3^
	Std	5.4950 × 10^1^	5.9934 × 10^1^	5.4738 × 10^1^	5.3282 × 10^1^	4.8856 × 10^1^	1.2936 × 10^2^	6.9906 × 10^1^	**4.7098 × 10^1^**	5.4370 × 10^1^
	Rank	6	7	5	2	**1**	8	9	4	3
F11	Ave	2.7269 × 10^3^	2.7382 × 10^3^	2.7169 × 10^3^	2.7068 × 10^3^	**2.6000 × 10^3^**	2.7523 × 10^3^	2.8540 × 10^3^	2.6300 × 10^3^	2.6333 × 10^3^
	Std	1.5854 × 10^2^	1.4578 × 10^2^	1.3092 × 10^2^	1.2786 × 10^2^	**2.0678 × 10^−3^**	1.7687 × 10^2^	1.9942 × 10^2^	8.2672 × 10^1^	1.0283 × 10^2^
	Rank	6	7	5	3	4	8	9	2	**1**
F12	Ave	2.8638 × 10^3^	2.8869 × 10^3^	**2.8570 × 10^3^**	2.9751 × 10^3^	2.8656 × 10^3^	2.8722 × 10^3^	2.8676 × 10^3^	2.8632 × 10^3^	2.8633 × 10^3^
	Std	1.6314	2.9853 × 10^1^	3.0255	8.2560 × 10^1^	**1.0737**	1.4889 × 10^1^	5.7676	1.7218	1.7532
	Rank	4	8	**1**	9	5	6	7	2	3

**Table 5:** Experimental results of CEC2022(dim = 20).

Function	Metric	VPPSO	IAGWO	LSHADE_cnEpSin	LSHADE_SPACMA	CPO	BKA	NRBO	NGO	EH-NGO
F1	Ave	**6.0438 × 10^3^**	1.1675 × 10^4^	2.8398 × 10^4^	1.0981 × 10^4^	1.2614 × 10^4^	7.7617 × 10^3^	1.4332 × 10^4^	1.4657 × 10^4^	7.4194 × 10^3^
	Std	**2.4777 × 10^3^**	6.0187 × 10^3^	1.8987 × 10^4^	1.4980 × 10^4^	4.4423 × 10^3^	7.6447 × 10^3^	3.6456 × 10^3^	3.1922 × 10^3^	2.8842 × 10^3^
	Rank	**1**	5	9	2	6	3	7	8	4
F2	Ave	4.7606 × 10^2^	5.1223 × 10^2^	4.7355 × 10^2^	4.5702 × 10^2^	4.6101 × 10^2^	6.3814 × 10^2^	7.4244 × 10^2^	4.6713 × 10^2^	**4.4771 × 10^2^**
	Std	3.4882 × 10^1^	4.3493 × 10^1^	2.8269 × 10^1^	1.5578 × 10^1^	1.0097 × 10^1^	3.9977 × 10^2^	1.1205 × 10^2^	**9.3185**	1.8899 × 10^1^
	Rank	6	7	5	2	3	8	9	4	**1**
F3	Ave	6.2736 × 10^2^	6.0813 × 10^2^	6.1256 × 10^2^	6.0159 × 10^2^	**6.0035 × 10^2^**	6.5071 × 10^2^	6.5477 × 10^2^	6.1787 × 10^2^	6.0036 × 10^2^
	Std	1.1023 × 10^1^	6.7760	6.9308	1.7611	**1.6020 × 10^−1^**	1.0618 × 10^1^	1.2397 × 10^1^	1.0515 × 10^1^	3.1194 × 10^−1^
	Rank	7	4	5	3	2	8	9	6	**1**
F4	Ave	8.6313 × 10^2^	8.6994 × 10^2^	8.7812 × 10^2^	8.4590 × 10^2^	8.9732 × 10^2^	8.7966 × 10^2^	9.4073 × 10^2^	8.7037 × 10^2^	**8.4342 × 10^2^**
	Std	1.5649 × 10^1^	1.3718 × 10^1^	1.8200 × 10^1^	1.0669 × 10^1^	1.3000 × 10^1^	2.9768 × 10^1^	2.0912 × 10^1^	1.3311 × 10^1^	**9.7284**
	Rank	3	5	7	2	8	6	9	4	**1**
F5	Ave	1.5418 × 10^3^	1.9917 × 10^3^	1.7171 × 10^3^	1.1209 × 10^3^	**9.4091 × 10^2^**	2.1513 × 10^3^	2.4452 × 10^3^	1.5592 × 10^3^	1.0929 × 10^3^
	Std	3.5550 × 10^2^	4.2000 × 10^2^	7.2714 × 10^2^	2.8491 × 10^2^	**9.4703 × 10^1^**	4.0987 × 10^2^	5.7340 × 10^2^	1.9234 × 10^2^	2.5990 × 10^2^
	Rank	4	7	6	2	**1**	8	9	5	3
F6	Ave	4.3600 × 10^3^	4.7503 × 10^3^	6.3902 × 10^3^	**3.1636 × 10^3^**	1.8285 × 10^4^	2.2125 × 10^6^	3.0212 × 10^7^	3.6871 × 10^3^	7.2207 × 10^3^
	Std	2.8003 × 10^3^	3.7289 × 10^3^	5.8277 × 10^3^	1.5740 × 10^3^	9.9958 × 10^3^	1.2046 × 10^7^	2.4369 × 10^7^	**1.5140 × 10^3^**	5.6961 × 10^3^
	Rank	4	3	5	**1**	8	7	9	2	6
F7	Ave	2.0927 × 10^3^	2.0821 × 10^3^	2.0968 × 10^3^	2.2007 × 10^3^	2.0645 × 10^3^	2.1178 × 10^3^	2.1958 × 10^3^	2.0918 × 10^3^	**2.0397 × 10^3^**
	Std	3.9836 × 10^1^	5.3556 × 10^1^	3.4648 × 10^1^	1.0231 × 10^2^	1.1618 × 10^1^	4.0574 × 10^1^	5.5731 × 10^1^	1.5917 × 10^1^	**9.6104**
	Rank	4	3	5	8	2	7	9	6	**1**
F8	Ave	2.2535 × 10^3^	2.2514 × 10^3^	2.2592 × 10^3^	2.3330 × 10^3^	2.2316 × 10^3^	2.2980 × 10^3^	2.3038 × 10^3^	2.2306 × 10^3^	**2.2232 × 10^3^**
	Std	4.5753 × 10^1^	5.1375 × 10^1^	4.9703 × 10^1^	1.9131 × 10^2^	**1.5559**	1.0902 × 10^2^	6.8411 × 10^1^	3.4825	3.9673
	Rank	4	2	6	8	5	7	9	3	**1**
F9	Ave	2.5004 × 10^3^	2.4973 × 10^3^	2.4861 × 10^3^	2.4813 × 10^3^	2.4817 × 10^3^	2.5448 × 10^3^	2.6265 × 10^3^	2.4808 × 10^3^	**2.4808 × 10^3^**
	Std	2.0720 × 10^1^	1.7363 × 10^1^	1.0228 × 10^1^	7.3796 × 10^−1^	4.3314 × 10^−1^	9.8474 × 10^1^	5.1206 × 10^1^	3.1439 × 10^−2^	**1.0961 × 10^−4^**
	Rank	7	6	5	3	4	8	9	2	**1**
F10	Ave	3.2898 × 10^3^	2.8082 × 10^3^	3.0493 × 10^3^	2.5440 × 10^3^	2.5332 × 10^3^	4.2748 × 10^3^	5.0691 × 10^3^	2.7487 × 10^3^	**2.5159 × 10^3^**
	Std	1.0167 × 10^3^	2.7091 × 10^2^	5.3361 × 10^2^	8.0438 × 10^1^	7.3769 × 10^1^	1.1090 × 10^3^	1.5594 × 10^3^	6.4537 × 10^2^	**5.7748 × 10^1^**
	Rank	5	6	7	3	2	8	9	4	**1**
F11	Ave	2.9695 × 10^3^	3.0295 × 10^3^	3.0682 × 10^3^	**2.8947 × 10^3^**	2.9192 × 10^3^	4.2329 × 10^3^	4.6442 × 10^3^	2.9392 × 10^3^	2.8967 × 10^3^
	Std	1.7842 × 10^2^	6.3888 × 10^2^	1.5551 × 10^2^	1.0887 × 10^2^	**7.5681 × 10^1^**	1.2338 × 10^3^	6.3383 × 10^2^	1.5642 × 10^2^	1.0980 × 10^2^
	Rank	2	6	7	3	4	8	9	5	**1**
F12	Ave	2.9896 × 10^3^	2.9400 × 10^3^	**2.9197 × 10^3^**	3.8624 × 10^3^	2.9840 × 10^3^	3.0730 × 10^3^	3.0734 × 10^3^	2.9523 × 10^3^	2.9471 × 10^3^
	Std	3.4895 × 10^1^	8.7523 × 10^1^	1.3095 × 10^1^	4.8950 × 10^2^	1.2838 × 10^1^	9.2981 × 10^1^	1.1533 × 10^2^	8.9661	**8.6687**
	Rank	6	2	**1**	9	5	7	8	4	3

**Table 6:** Friedman mean rank test result.

Suites	CEC2017		CEC2022			
Dimensions	30		10		20	
Algorithms	M.R	T.R	M.R	T.R	M.R	T.R
VPPSO	5.37	7	6.08	7	4.42	4.5
IAGWO	5.20	6	5.92	6	4.67	6
LSHADE_cnEpSin	3.33	2	3.42	2	5.67	7
LSHADE_SPACMA	4.37	4	4.92	5	3.83	2
CPO	4.33	3	3.67	4	4.17	3
BKA	7.27	8	6.83	8	7.08	8
NRBO	8.60	9	8.50	9	8.75	9
NGO	4.73	5	3.58	3	4.42	4.5
EN-MGO	1.80	1	2.08	1	2.00	1

**Table 7:** The description of datasets used in the comparative study.

ID	Name	Number of Features	Number of Instances	Number of Classes
Dataset 1	Tic-Tac-Toe Endgame	90	958	2
Dataset 2	BreastCancer Wisconsin (Original)	10	699	2
Dataset 3	Statlog (Heart)	13	270	2
Dataset 4	Wine	13	178	3
Dataset 5	Congressional Voting Records	16	435	2
Dataset 6	Zoo	16	101	7
Dataset 7	Lymphography	18	148	4
Dataset 8	Hepatitis	19	155	2
Dataset 9	German Credit Dataset Analysis	20	1000	2
Dataset 10	Waveform	21	5000	3
Dataset 11	Breast Cancer Wisconsin (Diagnostic)	30	569	2
Dataset 12	Ionosphere	34	351	2
Dataset 13	Dermatology	34	366	6
Dataset 14	Soybean (Small)	35	47	4
Dataset 15	Lung Cancer	56	32	3
Dataset 16	Connectionist Bench (Sonar, Mines vs. Rocks)	60	208	2
Dataset 17	Hill-Valley	100	1212	2
Dataset 18	Musk Version 1	166	476	2
Dataset 19	Semeion Handwritten Digit	265	1593	2
Dataset 20	Malware Executable Detection	531	373	2
Dataset 21	Parkinson’s Disease Classification	754	756	2
Dataset 22	CNA × 10^−9^	856	1080	3

**Table 8:** Comparison of fitness results of EH-NGO and other algorithms on 22 Datasets.

Function	Metric	VPPSO	IAGWO	LSHADE_cnEpSin	LSHADE_SPACMA	CPO	BKA	NRBO	NGO	EH-NGO
Dataset 1	Ave	**1.2463 × 10^−1^**	1.4901 × 10^−1^	1.2587 × 10^−1^	1.2836 × 10^−1^	1.5169 × 10^−1^	**1.2463 × 10^−1^**	1.4367 × 10^−1^	1.2836 × 10^−1^	**1.2463 × 10^−1^**
	Std	**8.4690 × 10^−17^**	2.2917 × 10^−2^	6.7990 × 10^−3^	1.1363 × 10^−2^	2.0297 × 10^−2^	**8.4690 × 10^−17^**	1.9499 × 10^−2^	1.1363 × 10^−2^	**8.4690 × 10^−17^**
Dataset 2	Ave	3.9449 × 10^−3^	3.9449 × 10^−3^	**3.0000 × 10^−3^**	4.4232 × 10^−3^	3.5783 × 10^−3^	5.6246 × 10^−3^	3.7449 × 10^−3^	**3.0000 × 10^−3^**	**3.0000 × 10^−3^**
	Std	2.6592 × 10^−3^	2.6332 × 10^−3^	**1.3233 × 10^−18^**	3.6244 × 10^−3^	2.6316 × 10^−3^	5.3744 × 10^−3^	2.6210 × 10^−3^	**1.3233 × 10^−18^**	**1.3233 × 10^−18^**
Dataset 3	Ave	8.4453 × 10^−2^	9.4231 × 10^−2^	7.6812 × 10^−2^	9.3538 × 10^−2^	8.1778 × 10^−2^	1.0077 × 10^−1^	7.8573 × 10^−2^	7.6838 × 10^−2^	**7.5641 × 10^−2^**
	Std	1.5532 × 10^−2^	2.2854 × 10^−2^	6.4135 × 10^−3^	1.7797 × 10^−2^	1.3414 × 10^−2^	1.8835 × 10^−2^	8.9898 × 10^−3^	6.4102 × 10^−3^	**0.0000**
Dataset 4	Ave	1.5897 × 10^−3^	1.8205 × 10^−3^	**1.5385 × 10^−3^**	1.6667 × 10^−3^	1.5897 × 10^−3^	1.6923 × 10^−3^	1.7436 × 10^−3^	**1.5385 × 10^−3^**	**1.5385 × 10^−3^**
	Std	1.9516 × 10^−4^	5.5261 × 10^−4^	**8.8219 × 10^−19^**	3.5472 × 10^−4^	1.9516 × 10^−4^	3.1295 × 10^−4^	3.4598 × 10^−4^	**8.8219 × 10^−19^**	**8.8219 × 10^−19^**
Dataset 5	Ave	2.3542 × 10^−3^	4.5557 × 10^−3^	**1.8750 × 10^−3^**	2.7500 × 10^−3^	2.8299 × 10^−3^	3.6633 × 10^−3^	3.0625 × 10^−3^	1.9375 × 10^−3^	**1.8750 × 10^−3^**
	Std	7.2819 × 10^−4^	5.2527 × 10^−3^	**1.3233 × 10^−18^**	9.0794 × 10^−4^	3.9709 × 10^−3^	4.0350 × 10^−3^	8.4253 × 10^−4^	2.5161 × 10^−4^	**1.3233 × 10^−18^**
Dataset 6	Ave	2.6042 × 10^−3^	3.1950 × 10^−2^	5.7375 × 10^−3^	2.9375 × 10^−3^	1.8729 × 10^−2^	3.0625 × 10^−3^	1.2629 × 10^−2^	2.5625 × 10^−3^	**2.5000 × 10^−3^**
	Std	2.3691 × 10^−4^	4.5067 × 10^−2^	1.7733 × 10^−2^	5.2291 × 10^−4^	3.6797 × 10^−2^	7.7595 × 10^−4^	2.9569 × 10^−2^	1.9071 × 10^−4^	**8.8219 × 10^−19^**
Dataset 7	Ave	2.1968 × 10^−2^	4.7304 × 10^−2^	2.8148 × 10^−3^	3.5426 × 10^−2^	3.0582 × 10^−2^	4.5003 × 10^−2^	3.1230 × 10^−2^	1.2280 × 10^−2^	**2.7963 × 10^−3^**
	Std	3.0976 × 10^−2^	3.4154 × 10^−2^	1.4095 × 10^−4^	3.4976 × 10^−2^	3.4522 × 10^−2^	3.4510 × 10^−2^	3.4795 × 10^−2^	2.3766 × 10^−2^	**1.0143 × 10^−4^**
Dataset 8	Ave	**6.7053 × 10^−2^**	6.7123 × 10^−2^	**6.7053 × 10^−2^**	6.7123 × 10^−2^	6.7088 × 10^−2^	6.7175 × 10^−2^	6.7526 × 10^−2^	**6.7053 × 10^−2^**	**6.7053 × 10^−2^**
	Std	**5.6460 × 10^−17^**	2.6706 × 10^−4^	**5.6460 × 10^−17^**	2.2851 × 10^−4^	1.3353 × 10^−4^	5.1128 × 10^−4^	2.8828 × 10^−4^	**5.6460 × 10^−17^**	**5.6460 × 10^−17^**
Dataset 9	Ave	2.1709 × 10^−1^	2.2213 × 10^−1^	1.9451 × 10^−1^	2.1777 × 10^−1^	2.0202 × 10^−1^	2.2970 × 10^−1^	2.1048 × 10^−1^	2.0054 × 10^−1^	**1.9350 × 10^−1^**
	Std	1.5478 × 10^−2^	1.8485 × 10^−2^	6.4343 × 10^−3^	1.6187 × 10^−2^	1.2000 × 10^−2^	1.1369 × 10^−2^	1.4625 × 10^−2^	1.0883 × 10^−2^	**5.7902 × 10^−3^**
Dataset 10	Ave	1.4135 × 10^−1^	1.4742 × 10^−1^	1.3374 × 10^−1^	1.4819 × 10^−1^	1.3717 × 10^−1^	1.5101 × 10^−1^	1.4260 × 10^−1^	1.3686 × 10^−1^	**1.3369 × 10^−1^**
	Std	7.3036 × 10^−3^	9.5755 × 10^−3^	3.0429 × 10^−3^	6.8011 × 10^−3^	7.0358 × 10^−3^	7.4064 × 10^−3^	7.5487 × 10^−3^	4.7890 × 10^−3^	**2.3663 × 10^−3^**
Dataset 11	Ave	2.2237 × 10^−2^	4.3640 × 10^−2^	2.2025 × 10^−2^	1.2074 × 10^−2^	2.0258 × 10^−2^	2.4994 × 10^−2^	4.7097 × 10^−2^	2.6901 × 10^−3^	**2.5345 × 10^−3^**
	Std	2.1956 × 10^−2^	1.5695 × 10^−2^	1.5643 × 10^−2^	1.4229 × 10^−2^	2.0904 × 10^−2^	1.4974 × 10^−2^	1.4524 × 10^−2^	5.3653 × 10^−3^	**5.3225 × 10^−3^**
Dataset 12	Ave	5.0361 × 10^−3^	1.1154 × 10^−2^	**9.7059 × 10^−4^**	1.3431 × 10^−3^	1.0980 × 10^−3^	6.0476 × 10^−3^	1.1723 × 10^−2^	9.9020 × 10^−4^	1.1471 × 10^−3^
	Std	9.7187 × 10^−3^	1.3555 × 10^−2^	**1.3709 × 10^−4^**	4.2082 × 10^−4^	2.4344 × 10^−4^	1.0684 × 10^−2^	1.3532 × 10^−2^	1.4416 × 10^−4^	1.9464 × 10^−4^
Dataset 13	Ave	2.0294 × 10^−3^	7.0833 × 10^−3^	1.6765 × 10^−3^	2.4216 × 10^−3^	1.9902 × 10^−3^	3.3676 × 10^−3^	3.7990 × 10^−3^	1.8431 × 10^−3^	**1.5294 × 10^−3^**
	Std	3.9649 × 10^−4^	1.0206 × 10^−2^	2.5791 × 10^−4^	5.7637 × 10^−4^	3.1548 × 10^−4^	4.9279 × 10^−3^	4.8985 × 10^−3^	3.6989 × 10^−4^	**1.6202 × 10^−4^**
Dataset 14	Ave	6.1905 × 10^−4^	8.0000 × 10^−4^	**5.7143 × 10^−4^**	5.9048 × 10^−4^	**5.7143 × 10^−4^**	5.9048 × 10^−4^	1.4286 × 10^−3^	**5.7143 × 10^−4^**	**5.7143 × 10^−4^**
	Std	1.0830 × 10^−4^	2.7466 × 10^−4^	**0.0000**	7.2488 × 10^−5^	**0.0000**	7.2488 × 10^−5^	4.8044 × 10^−4^	**0.0000**	**0.0000**
Dataset 15	Ave	4.7619 × 10^−4^	1.2179 × 10^−2^	**3.5714 × 10^−4^**	4.0476 × 10^−4^	3.8095 × 10^−4^	5.0595 × 10^−4^	2.0774 × 10^−3^	3.6905 × 10^−4^	**3.5714 × 10^−4^**
	Std	2.2162 × 10^−4^	6.0132 × 10^−2^	**2.2055 × 10^−19^**	1.6200 × 10^−4^	6.1740 × 10^−5^	2.3487 × 10^−4^	5.9408 × 10^−4^	4.5305 × 10^−5^	**2.2055 × 10^−19^**
Dataset 16	Ave	1.1444 × 10^−3^	1.0428 × 10^−2^	1.2111 × 10^−3^	2.9111 × 10^−3^	1.4889 × 10^−3^	6.2833 × 10^−3^	7.9833 × 10^−3^	8.0000 × 10^−4^	**5.7222 × 10^−4^**
	Std	4.4793 × 10^−4^	1.8849 × 10^−2^	**2.5496 × 10^−4^**	8.9816 × 10^−3^	3.6602 × 10^−4^	1.5093 × 10^−2^	1.5005 × 10^−2^	2.7572 × 10^−4^	2.6767 × 10^−4^
Dataset 17	Ave	3.2761 × 10^−1^	3.4022 × 10^−1^	3.2139 × 10^−1^	3.0567 × 10^−1^	3.3500 × 10^−1^	3.3254 × 10^−1^	3.3919 × 10^−1^	2.9695 × 10^−1^	**2.8244 × 10^−1^**
	Std	2.1010 × 10^−2^	2.2605 × 10^−2^	**1.0709 × 10^−2^**	2.0204 × 10^−2^	1.5539 × 10^−2^	2.0813 × 10^−2^	1.4667 × 10^−2^	1.4801 × 10^−2^	1.5694 × 10^−2^
Dataset 18	Ave	1.6687 × 10^−3^	5.0984 × 10^−3^	2.0261 × 10^−3^	2.1988 × 10^−3^	2.2751 × 10^−3^	2.0020 × 10^−3^	4.3648 × 10^−3^	1.6406 × 10^−3^	**1.1787 × 10^−3^**
	Std	6.1964 × 10^−4^	6.4308 × 10^−3^	**2.2232 × 10^−4^**	7.1729 × 10^−4^	3.0417 × 10^−4^	8.0848 × 10^−4^	3.9005 × 10^−3^	3.2755 × 10^−4^	2.7432 × 10^−4^
Dataset 19	Ave	1.4712 × 10^−2^	1.9815 × 10^−2^	1.1263 × 10^−2^	1.6297 × 10^−2^	1.2769 × 10^−2^	1.5508 × 10^−2^	1.7829 × 10^−2^	1.2348 × 10^−2^	**9.3409 × 10^−3^**
	Std	4.1980 × 10^−3^	4.9971 × 10^−3^	2.4294 × 10^−3^	4.4965 × 10^−3^	2.9123 × 10^−3^	3.0924 × 10^−3^	4.1523 × 10^−3^	3.2359 × 10^−3^	**2.4236 × 10^−3^**
Dataset 20	Ave	1.2492 × 10^−4^	1.4444 × 10^−3^	5.9636 × 10^−5^	4.3315 × 10^−5^	8.4746 × 10^−5^	6.7797 × 10^−5^	3.5342 × 10^−3^	5.1475 × 10^−5^	**3.0760 × 10^−5^**
	Std	6.3235 × 10^−5^	1.3073 × 10^−3^	2.0441 × 10^−5^	2.6240 × 10^−5^	3.1571 × 10^−5^	3.5526 × 10^−5^	1.5437 × 10^−4^	1.6013 × 10^−5^	**1.4782 × 10^−5^**
Dataset 21	Ave	1.1254 × 10^−1^	2.2330 × 10^−1^	1.4696 × 10^−1^	1.1384 × 10^−1^	1.1048 × 10^−1^	1.3306 × 10^−1^	2.0542 × 10^−1^	9.3752 × 10^−2^	**8.6927 × 10^−2^**
	Std	3.1315 × 10^−2^	3.2728 × 10^−2^	3.0113 × 10^−2^	1.7036 × 10^−2^	2.6906 × 10^−2^	2.5382 × 10^−2^	5.6475 × 10^−2^	1.3830 × 10^−2^	**9.4542 × 10^−3^**
Dataset 22	Ave	3.7466 × 10^−2^	5.9247 × 10^−2^	2.6920 × 10^−2^	3.3926 × 10^−2^	3.2631 × 10^−2^	4.5683 × 10^−2^	4.2879 × 10^−2^	2.7142 × 10^−2^	**2.3445 × 10^−2^**
	Std	8.7821 × 10^−3^	1.6688 × 10^−2^	8.7211 × 10^−3^	8.1027 × 10^−3^	7.6160 × 10^−3^	1.0290 × 10^−2^	1.1613 × 10^−2^	8.8448 × 10^−3^	**6.3979 × 10^−3^**
Mean_rank		3.93	5.76	4.14	5.25	5.12	6.11	7.46	4.93	**1.9**
Rank		2	7	3	6	5	8	9	4	**1**

**Table 9:** Comparison of classification accuracy results of EH-NGO and other algorithms on 22 Datasets.

Function	Metric	VPPSO	IAGWO	LSHADE_cnEpSin	LSHADE_SPACMA	CPO	BKA	NRBO	NGO	EH-NGO
Dataset 1	Ave	**88.42%**	85.72%	88.28%	88.00%	85.40%	**88.42%**	86.28%	88.00%	**88.42%**
Dataset 2	Ave	99.95%	99.95%	**100.00%**	99.90%	99.95%	99.76%	99.95%	**100.00%**	**100.00%**
Dataset 3	Ave	91.73%	90.74%	92.47%	90.74%	91.98%	90.00%	92.35%	92.47%	**92.59%**
Dataset 4	Ave	**100.00%**	**100.00%**	**100.00%**	**100.00%**	**100.00%**	**100.00%**	**100.00%**	**100.00%**	**100.00%**
Dataset 5	Ave	**100.00%**	99.84%	**100.00%**	**100.00%**	99.92%	99.92%	**100.00%**	**100.00%**	**100.00%**
Dataset 6	Ave	**100.00%**	97.00%	99.67%	**100.00%**	98.33%	**100.00%**	99.00%	**100.00%**	**100.00%**
Dataset 7	Ave	98.10%	95.48%	**100.00%**	96.67%	97.14%	95.71%	97.14%	99.05%	**100.00%**
Dataset 8	Ave	**93.33%**	**93.33%**	**93.33%**	**93.33%**	**93.33%**	**93.33%**	**93.33%**	**93.33%**	**93.33%**
Dataset 9	Ave	78.43%	77.93%	**80.83%**	78.40%	80.00%	77.13%	79.17%	80.10%	**80.83%**
Dataset 10	Ave	86.53%	85.81%	87.24%	85.83%	86.89%	85.58%	86.33%	86.92%	**87.27%**
Dataset 11	Ave	97.86%	95.71%	97.86%	98.87%	98.04%	97.56%	95.42%	**99.82%**	**99.82%**
Dataset 12	Ave	99.62%	99.05%	**100.00%**	**100.00%**	**100.00%**	99.52%	99.05%	**100.00%**	**100.00%**
Dataset 13	Ave	**100.00%**	99.54%	**100.00%**	**100.00%**	**100.00%**	99.91%	99.91%	**100.00%**	**100.00%**
Dataset 14	Ave	**100.00%**	**100.00%**	**100.00%**	**100.00%**	**100.00%**	**100.00%**	**100.00%**	**100.00%**	**100.00%**
Dataset 15	Ave	**100.00%**	98.89%	**100.00%**	**100.00%**	**100.00%**	**100.00%**	**100.00%**	**100.00%**	**100.00%**
Dataset 16	Ave	**100.00%**	99.17%	**100.00%**	99.83%	**100.00%**	99.50%	99.50%	**100.00%**	**100.00%**
Dataset 17	Ave	67.13%	66.03%	67.82%	69.31%	66.45%	66.53%	66.20%	70.08%	**71.65%**
Dataset 18	Ave	**100.00%**	99.79%	**100.00%**	**100.00%**	**100.00%**	**100.00%**	99.93%	**100.00%**	**100.00%**
Dataset 19	98.81%	98.36%	99.16%	98.68%	99.06%	98.72%	98.62%	99.06%	**99.29%**	98.81%
Dataset 20	**100.00%**	**100.00%**	**100.00%**	**100.00%**	**100.00%**	**100.00%**	**100.00%**	**100.00%**	**100.00%**	**100.00%**
Dataset 21	88.76%	77.69%	85.29%	88.53%	88.89%	86.58%	79.64%	90.58%	**91.47%**	88.76%
Dataset 22	96.91%	94.48%	97.75%	97.13%	97.25%	96.08%	96.14%	97.75%	**98.12%**	96.91%
Mean_rank		3.93	5.76	4.14	5.25	5.12	6.11	7.46	4.93	**1.9**
Rank		2	7	3	6	5	8	9	4	**1**

**Table 10:** Comparison of average number of selected features of EH-NGO and other algorithms on 22 Datasets.

Function	Metric	VPPSO	IAGWO	LSHADE_cnEpSin	LSHADE_SPACMA	CPO	BKA	NRBO	NGO	EH-NGO
Dataset 1	Ave	9.00	6.87	8.87	8.60	**6.47**	9.00	7.07	8.60	9.00
Dataset 2	Ave	3.47	3.47	**3.00**	3.47	3.10	3.23	3.27	**3.00**	**3.00**
Dataset 3	Ave	3.33	3.33	2.93	2.43	3.03	**2.30**	3.63	2.97	3.00
Dataset 4	Ave	2.07	2.37	**2.00**	2.17	2.07	2.20	2.27	**2.00**	**2.00**
Dataset 5	Ave	3.77	4.83	**3.00**	4.40	3.30	4.63	4.90	3.10	**3.00**
Dataset 6	Ave	4.17	3.60	3.90	4.70	4.00	4.90	4.37	4.10	**3.57**
Dataset 7	Ave	5.60	4.53	5.07	4.37	5.03	4.63	5.30	5.13	**4.13**
Dataset 8	Ave	**2.00**	2.13	**2.00**	2.13	2.07	2.23	2.90	**2.00**	**2.00**
Dataset 9	Ave	7.17	7.33	7.50	7.87	8.03	**6.63**	8.47	7.07	**6.63**
Dataset 10	Ave	16.87	**14.63**	15.57	16.53	15.43	17.33	15.33	15.47	16.17
Dataset 11	Ave	3.07	3.63	2.43	2.63	2.43	2.50	5.17	2.77	**2.30**
Dataset 12	Ave	4.30	5.87	**3.30**	4.57	3.73	4.53	7.80	3.37	3.90
Dataset 13	Ave	6.90	8.50	5.70	8.23	6.77	8.33	9.80	6.27	**5.20**
Dataset 14	Ave	2.17	2.80	**2.00**	2.07	**2.00**	2.07	5.00	**2.00**	**2.00**
Dataset 15	Ave	2.67	6.60	**2.00**	2.27	2.13	2.83	11.63	**2.00**	**2.00**
Dataset 16	Ave	6.87	13.07	7.27	7.57	8.93	8.00	18.20	4.80	**3.43**
Dataset 17	Ave	22.50	39.43	28.43	18.57	28.17	11.77	45.57	17.63	**11.00**
Dataset 18	Ave	27.70	49.67	33.63	36.50	37.77	33.23	60.80	27.23	**19.57**
Dataset 19	Ave	76.37	96.10	78.47	85.37	90.87	75.47	109.47	79.73	**60.53**
Dataset 20	Ave	6.63	76.70	3.17	2.30	4.50	3.60	187.67	2.73	**1.63**
Dataset 21	Ave	91.70	182.30	99.53	23.80	36.37	184.53	293.97	35.57	**13.50**
Dataset 22	Ave	591.50	395.03	411.43	471.60	465.37	588.67	401.03	413.97	**389.70**
Mean_ features		7.42	10.15	7.70	7.73	8.03	7.24	12.30	6.64	**5.66**
Rank		4	8	5	6	7	3	9	2	**1**

## Data Availability

All data in this paper are included in the manuscript.
